# Effect of Agricultural Organic Inputs on Nanoplastics Transport in Saturated Goethite-Coated Porous Media: Particle Size Selectivity and Role of Dissolved Organic Matter

**DOI:** 10.1021/acs.est.1c07574

**Published:** 2022-02-28

**Authors:** Jie Ma, Yan Qiu, Junying Zhao, Xiaoxue Ouyang, Yujie Zhao, Liping Weng, Arafat MD Yasir, Yali Chen, Yongtao Li

**Affiliations:** † Key Laboratory for Environmental Factors Control of Agro-Product Quality Safety, Ministry of Agriculture and Rural Affairs, Tianjin, 300191, China; ‡ Agro-Environmental Protection Institute, Ministry of Agriculture and Rural Affairs, Tianjin 300191, China; § School of Environmental Science and Safety Engineering, Tianjin University of Technology, Tianjin, 300384, China; ∥ Department of Soil Quality, Wageningen University, Wageningen 6700 HB, The Netherlands; ⊥ College of Resource and Environmental Engineering, Jiangxi University of Science and Technology, Ganzhou Jiangxi 341000, China; # College of Natural Resources and Environment, South China Agricultural University, Guangzhou, 510642, China

**Keywords:** nanoplastics, dissolved organic matter, particle size, humic acid, cellulose

## Abstract

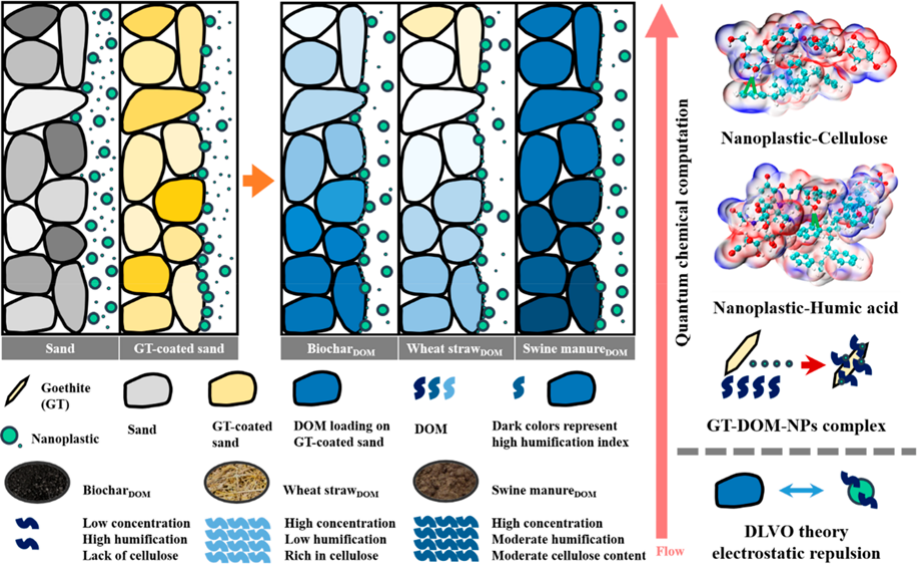

The transport of nanoplastics (NPs) through porous media is influenced by dissolved organic matter (DOM) released from agricultural organic inputs. Here, cotransport of NPs with three types of DOM (biochar_DOM_ (BC_DOM_), wheat straw_DOM_ (WS_DOM_), and swine manure_DOM_ (SM_DOM_)) was investigated in saturated goethite (GT)-coated sand columns. The results showed that codeposition of 50 nm NPs (50NPs) with DOM occurred due to the formation of a GT–DOM–50NPs complex, while DOM loaded on GT-coated sand and 400 nm NPs (400NPs) aided 400NPs transport due to electrostatic repulsion. According to the quantum chemical calculation, humic acid and cellulose played a significant role in 50NPs retardation. Owing to its high concentration, moderate humification index (HIX), and cellulose content, SM_DOM_ exhibited the highest retardation of 50NPs transport and promoting effect on 400NPs transport. Owing to a high HIX, the effect of BC_DOM_ on the mobility of 400NPs was higher than that of WS_DOM_. However, high cellulose content in WS_DOM_ caused it to exhibit a 50NPs retardation ability that was similar to that of BC_DOM_. Our results highlight the particle size selectivity and significant influence of DOM type on the transport of NPs and elucidate their quantum and colloidal chemical-interface mechanisms in a typical agricultural environment.

## Abstract 2

Agricultural organic input-derived DOM retarded 50 nm nanoplastic transport by forming GT−DOM−50NPs and promoted 400 nm nanoplastic transport by DLVO repulsion.

## Introduction

The environmental behavior and biological toxicity of microplastics (MPs) in water have become a widespread concern.^1,2^ Moreover, inputs from agricultural film crushing, organic fertilization, sewage irrigation, agricultural sludge production, atmospheric precipitation, and surface runoff can introduce MPs into farmlands.^3−6^ A previous study reported that the annual input of MPs to farmland soil greatly exceeds that to the ocean.^7^ In the natural environment, MPs can be mechanically broken, chemically decomposed, and biodegraded into nanoplastics (NPs),^1,8,9^ which exhibit environmental behaviors differing from those of MPs due to their smaller particle size^10^ and specific biological toxicity;^11^ thus, NPs must be urgently investigated to understand their environmental fate.

Several studies have reported the behaviors of NPs transport,^10,12−17^ for which the size of NPs is considered as an important influencing factor. For example, large-sized plastics have high retardation in sand due to the formation of small barrier or agglomeration with sand at a salinity of 35 PSU.^10^ Similarly, in a previous study, MP fibers exhibited low detachment from sludge and reduced mobility through the column, while cotransport between the mobile organic fraction and NPs was 50%.^18^ The dispersivity for nanoparticles is not only particle-size dependent, but also a property of the porous medium.^19^ Although changes in the surface properties or deposition sites on porous medium have enhanced the deposition of large-sized NPs (200 and 2000 nm) in the presence of iron oxide, they have not influenced the transport of small-sized NPs (20 nm).^13^ Similarly, the retention of NPs (100 nm) in different soils is positively correlated with the Fe/Al oxide content.^12^ Furthermore, Fe mineral colloids and NPs can cause heteroaggregation.^20^ Thus, Fe minerals play a crucial role in size differentiation during the transport of NPs.

Organic inputs, such as biochar, straw, and livestock manure, applied to agricultural soil affect soil ecological processes and the fate of pollutants, including that of NPs. These inputs are sustainable, environmentally friendly, economical fertilizers that can increase crop yield^21^ and effectively improve soil quality by increasing soil organic carbon sequestration.^22−24^ Moreover, dissolved organic matter (DOM) released from organic inputs can readily adsorb on clay minerals, Fe oxides, and the surface of nanoparticles; hence, they significantly alter the surface chemistry and retention–repulsion properties of the water–sand–nanoparticle system. Therefore, DOM is a crucial substance in soils and can regulate the transport of nanoparticle and colloids.^18,20,25−32^ Furthermore, DOM from different sources exhibits complex composition and diversified characteristics. Our previous studies suggest that humification, size, and morphology of DOM or humic acid (HA) are essential for controlling the transport of ferrihydrite nanoparticles.^25,29,33^ For plastics, HA improves the stability of MPs,^34^ while organic matter and Fe mineral colloids promote NPs agglomeration via bridging or neutralization.^20,35^ The large particle size lead to low heteroaggregation rate of NPs with DOM, wherein 200 nm NPs show higher stability than 50 nm NPs.^14^ The opposing effects of DOM on the stability of NPs with different sizes confirm its crucial role. The effects of DOM on NPs mobility depend on the reactivities of both materials.^36^ The adsorptive interaction between DOM and NPs has been observed using a scanning electron microscopy (SEM).^35^ However, existing research is inadequate to clarify the microinterfacial mechanism of NPs under conditions resembling actual farmland soil, particularly after the application of organic input and in the presence of complex multicomponent DOM. Previous studies have reported that the cotransport of different particles is affected by several factors;^37−39^ therefore, the cotransport of NPs with DOM in porous media may also be variable and size-dependent. However, the microinterface mechanism affecting this cotransport has not been extensively studied in a typical agricultural environment; thus, NPs of different sizes cotransported with agricultural organic input-derived DOM should be further investigated.

In this study, the influence of DOM released from agricultural organic inputs (biochar, straw, and livestock manure) on the transport of NPs in porous media containing iron minerals was investigated, and the mechanism underlying the microinterfacial interaction among DOM, goethite, and NPs was elucidated via column experiments, transport simulation, SEM, DOM characterization, Derjaguin–Landau–Verwey–Overbeek (DLVO) theory, and quantum chemical calculations. The results of this study can improve our understanding of NPs mobility in agricultural environments.

## Materials and Methods

### Preparation and Characterization of Experimental Materials

Goethite (GT) was prepared according to the method described in Supporting Information (SI) S1. The purity of GT was confirmed using X-ray diffraction analysis, as described in our previous study.^27^ The BET specific surface area and the pH of zero charge were 87.65 m^2^·g^–1^ and 9.2, respectively.^27,40^

Biochar was produced from commercial wheat straw at 300 °C. Wheat straw and swine manure were collected from rural areas in Tianjin, China. The dry biochar, wheat straw, and swine manure were ground into powder, passed through 0.425 mm nylon sieves, and labeled as BC, SW, and SM, respectively. DOM from these three organic inputs was prepared by adding 1.0 g of BC, SW, or SM to 400 mL of Milli-Q water. The suspension was homogenized by stirring for 60 min and filtered through a 0.45-μm membrane to remove large suspended matter after settling. To simulate the DOM releasing ability of different agricultural organic inputs, the filtered solutions (BC_DOM_, WS_DOM_, and SM_DOM_) and their diluents (half and quarter) were directly used in the subsequent experiments. The concentrations and zeta potentials of BC_DOM_, WS_DOM_, and SM_DOM_ at pH 6.0, were measured using a total organic carbon (TOC) analyzer (Aurora 1030S, OI Analytical) and dynamic light scattering (DLS) analyzer (Zetasizer Nano ZS, Malvern), respectively. Steric exclusion-ultrahigh performance liquid chromatography (SEC-UPLC, Acquity H-Class, Waters) was used to detect the molecular weights of different DOM, and the corresponding calibration curves are shown in SI Figure S1. The fluorescence excitation–emission matrices (EEMs) of BC_DOM_, WS_DOM_, and SM_DOM_ were measured using a spectrofluorometer. (FluoroMax-4, Horiba). The method of humification index (HIX) calculation is shown in SI S2.

In this study, Polystyrene NP spheres suspensions (Huge Biotechnology Co., Ltd., China) with 50 nm (50NPs) and 400 nm (400NPs) diameters were used, and their influent concentrations were maintained at 200 and 50 mg L^–1^, respectively. The point of zero charge (pH_PZC_) of NPs was beyond the pH range of 3–10.^41^ The NPs and NPs–DOM concentrations were determined by measuring extinction at 300 nm with a spectrophotometer and using the linear calibration curves of the respective standard solutions (SI Figure S2). Zeta potentials of the NPs and NPs–DOM at pH 6.0 were determined using a DLS analyzer and are shown in SI Table S1.

### Transport Experiment

Transport experiments were performed in 10 cm-long and 1.5 cm-inner diameter glass chromatographic columns. Quartz sand particles of two sizes (70- and 338-μm average particle size) were cleaned by immersing in 6 M HCl, followed by repeated rinsing with Milli-Q water. Different amounts of sand and GT (0%–2%) were mixed with 10% Milli-Q water to adhere the material to the surface of the sand. The columns were wet packed with quartz sand or GT-coated sand. The effective porosity and bulk density of the 70-μm packed sand were 0.38 ± 0.02 cm^3^·cm^–3^ and 1.59 ± 0.04 g cm^–3^, respectively, and those of the 338-μm packed sand were 0.44 ± 0.03 cm^3^·cm^–3^ and 1.45 ± 0.07 g·cm^–3^, respectively. These values and experimental flow velocity (1 mL·min^–1^) are consistent with those reported in our previous studies.^25,42^ Therefore, the longitudinal dispersivities in this study were considered as 0.206 for the 70-μm sand and 0.487 for the 338-μm sand. Before the transport experiments, the columns were preconditioned with approximately 15 pore volumes (PVs) of 10 mM NaCl in Milli-Q water using a peristaltic pump (BT-100 1F, Longer) in the upflow mode. Generally, the rate of particle deposition for upflow mode was greater than that for downflow mode.^43^ All column experiments were performed at a typical soil pH (6.0), and 10 PVs of NPs, DOM, or NPs–DOM in 10 mM NaCl were injected into the columns, followed by elution with 5 PVs of 10 mM NaCl. Owing to the presence of ions in the DOM solution, NaCl was added to ensure the similarity of ionic strength under different conditions. The specific experimental conditions are shown in SI Table S3. The surface functional groups on the DOM mixed with NPs were analyzed using Fourier transform infrared (FTIR) spectroscopy (Nicolet iS5, Thermo) in the 400–4000 cm^–1^ range after freeze-drying.

In the transport experiments, the NPs concentrations in the effluent were measured at each PV using a spectrophotometer. For individual DOM transport, the concentrations of DOM in the effluent and DOM retention after the experiments were measured using a TOC analyzer and solid module, respectively. Transport experiment data were simulated using a one-dimensional nanoparticle transport model considering attachment/detachment and chemical nonequilibrium with two-site (reversible and irreversible) kinetic retention.^44^ Although the nanoparticle (reaction-limited and diffusion-limited) aggregation^45^ was not considered, the proposed model can reflect the characteristics of NPs transport. Details of the transport model are shown in SI S5. The interactions between NPs and the sand surfaces were elucidated based on the DLVO theory,^46,47^ which are provided in SI S6.

After the transport experiments, the column sand was divided into four 2.5 cm long layers. As NPs cannot be easily distinguished from their complex mixtures (NPs, GT, DOM, and sand), the its deposition in the column was not analyzed. However, the zeta potentials of the sand before and after cotransport experiments (finely ground) were measured using a DLS analyzer for DLVO analysis, and are shown in SI Tables S2 and S3. The surface elemental compositions of the inlet sand (0–2.5 cm) during the cotransport of 50NPs with different DOM were determined by X-ray photoelectron spectroscopy (XPS, ESCALAB 250XI, Thermo). Moreover, the deposition of NPs at the column inlet during cotransport with SM_DOM_ was analyzed using SEM with a Zeiss Merlin Compact (OxfordX-MAX, Zeiss).

### Quantum Chemical Calculation

To investigate the intricacies of NPs–DOM interactions, representatives of polysaccharides, lipid-like compounds, and proteins were selected. The basis for selecting typical DOM (cellulose (CL), amylose (AM), oleic acid (OA) tetrapeptide (TP, valine-glycine-serine-alanine), HA, and fulvic acid (FA)) and their molecular formula are shown in SI S7 and SI Figure S3. The original configurations of the complexes were searched using the Molclus program.^48^ The configurations were optimized based on Parameterized Model number 6 and the all-electron density functional theory was calculated using Gaussian 16^49^ (Details given in SI S7). The binding energy equations are presented in SI S8. To clarify the interaction mechanism, the electrostatic potential (ESP) was analyzed using Multiwfn software.^50^ The complex structures and contour surfaces of ESP were visualized using the Visual Molecular Dynamics software.^51^ Each system was divided into three key regions, and their area vertex coordinates and interpenetration distances were calculated.

## Results and Discussion

### Effect of Goethite on Nanoplastics Transport

**Figure 1:**
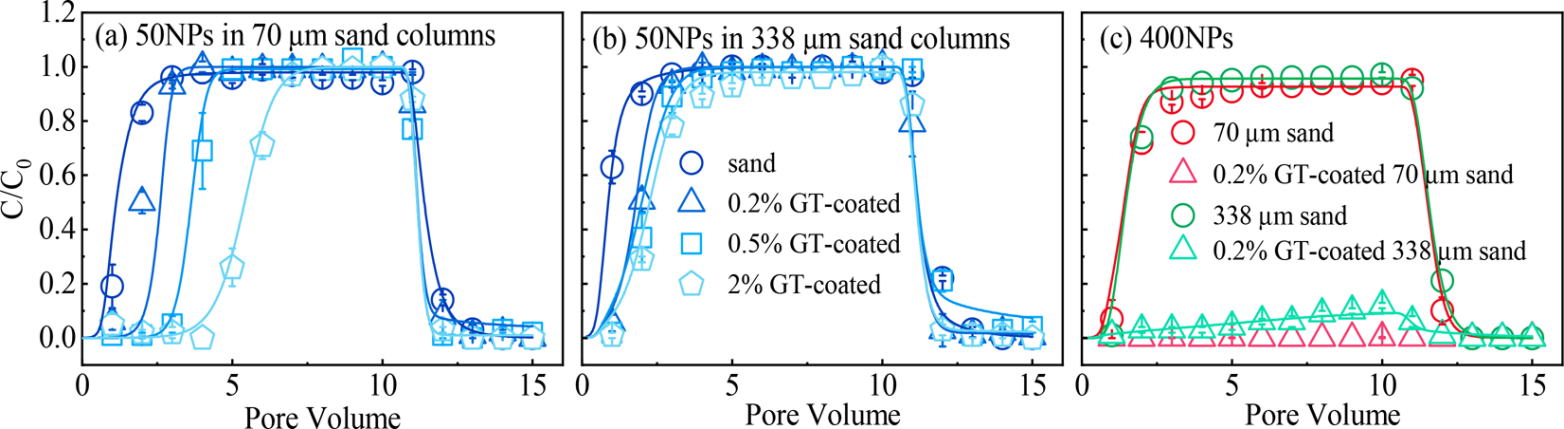
Breakthrough curves of the transport of 50NPs (a,b) and 400NPs (c) in the pure or GT-coated 70-μm and 338-μm sand columns at an influent pH of 6.0. Symbols indicate observed data and solid lines indicate simulation fitting. Recovery rates of <5% in the effluent did not fit.

The observed and simulated results of NPs transport with different sizes (50 and 400 nm) in sand or GT-coated sand columns are shown in Figure 1 and SI Table S4. Transport of both 50NPs and 400NPs was unimpeded in the 70- and 338-μm quartz columns (effluent recovery within 91.9%–106.6%) (Figure 1 and SI Table S4). The transport of 50NPs decreased with increasing GT content, which was more evident in fine sand, as indicated by a significantly reduced recovery rate (from 98.8% to 51.1%) (Figure 1a,b and SI Table S4). In a previous study, the presence of Fe minerals did not influence the transport of 20 nm NPs.^13^ However, in our study, as the NPs size and Fe mineral content increased, the effect of Fe minerals on decreasing NPs transport was observed. A previous study reported that high contents (15–45%) of Fe mineral caused greater retention of 50NPs at pH 5–9 than that in this study.^52^ Due to the unsaturated coordination, GT easily coordinated with water and formed a hydroxylated surface after water dissociation. The surface hydroxyl group of GT could undergo proton migration, showing amphoteric oxidation characteristics and finally resulting in NPs adsorption.^53^ Although GT caused the retardation of 50NPs, the retention was primarily in reversible sites, which corresponded to high *k*_1a_ and low *k*_2a_ values (SI Table S4). Reversible retention facilitated the maintenance of the BTC height at approximately one (Figure 1). A 0.2% GT content considerably retarded 400NPs transport in coarse and fine sand columns, suggesting that GT had a strong retardation effect on large NPs. Compared with that of 50NPs, the value of *k*_2a_ increased for 400NPs, indicating a more irreversible deposition for larger NPs. Similar observations were made by a study of cotransport and deposition of iron oxides with different-sized plastic particles.^13^ Additionally, the zeta potential of sand changed from negative to positive in the presence of GT (SI Table S2), decreasing the primary energy barrier of DLVO to below zero, regardless of the NPs size (SI Figure S4). These results were similar to a previous study, which reported a low primary energy barrier between NPs and a soil with high Fe content.^12^ Thus, the transport of 400NPs was retarded; however, the retardation of 50NPs was low with rare irreversible retention. This result indicates that the DLVO theory effectively explains the transport of large NPs; however, there is a knowledge gap between the DLVO theory and the transport of small NPs.

### Physicochemical Properties of DOM and DOM-Nanoplastics

**Figure 2:**
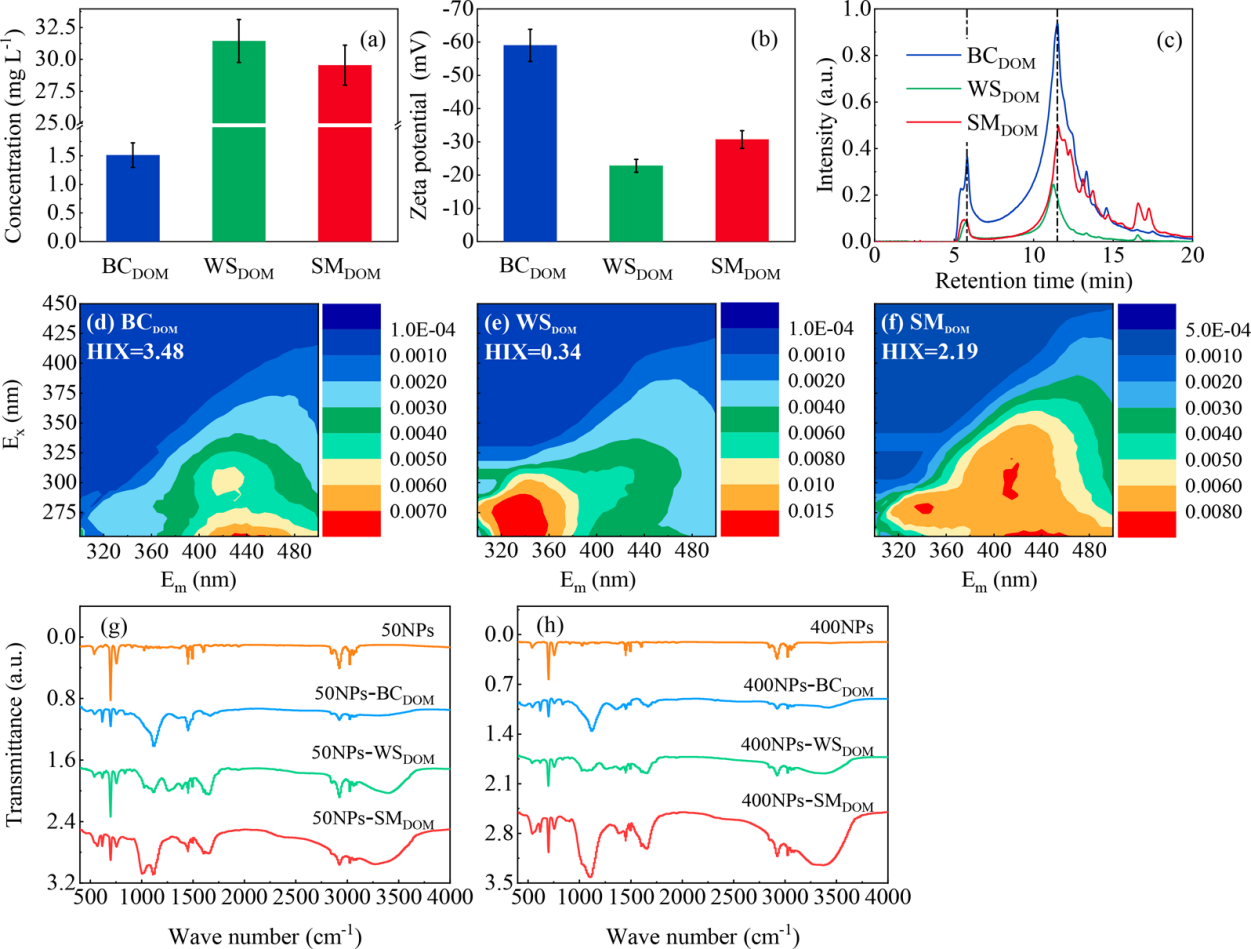
Concentrations (a), zeta potential (b), steric exclusion chromatography (c), excitation–emission spectra and humification indices for DOM released by different agricultural organic inputs (d–f), and FTIR spectra of NPs and different DOM mixed with 50NPs (g) and 400NPs (h).

The physicochemical properties of DOM released from different agricultural organic inputs are presented in Figure 2a–f. The DOM concentration released by BC (1.5 mg L^–1^) was lower than that released by WS (31.4 mg L^–1^) and SM (29.5 mg L^–1^) (Figure 2a), while the zeta potential of BC_DOM_ (−59.0 mV) was higher than that of WS_DOM_ (−22.8 mV) and SM_DOM_ (−30.7 mV) (Figure 2b), similar to the results of our previous study.^25^ The SEC-UPLC results revealed the typical size distribution of DOM. The first peak time of the three DOM species was similar (∼5.7 min), showing a similar molecular weight of 53.9 kDa (Figure 2c). The second peak of WS_DOM_ (∼11.2 min) appeared to be slightly earlier than those of BC_DOM_ and SM_DOM_ (∼11.7 min), showing a higher molecular weight of WS_DOM_ (1.1 kDa) than those of BC_DOM_ and SM_DOM_ (0.8 kDa) (Figure 2c).

The HIX of BC_DOM_ (3.48) was high because of the high fluorescence intensity of the HA-like area (region of *E*_*x*_ = 250–420 nm, *E*_m_ = 380–520 nm) (Figure 2d). The highlighted area of WS_DOM_ was similar to the EEM map of macrophyte-derived DOM,^54^ and the HIX of WS_DOM_ (0.34) was the lowest (Figure 2e). The degree of humification of SM_DOM_ (2.19) was between those of BC_DOM_ and WS_DOM_ (Figure 2f), and microbial byproducts and amino acids were the major fluorescent DOM in SM_DOM_.^55^

The FTIR spectra of NPs and their mixtures with different DOM showed no significant difference between 50NPs–DOM and 400NPs–DOM (Figure 2g,h). The series absorption peaks of NPs corresponded to the characteristic absorption peaks of polystyrene^41^ (Details given in SI S12). To eliminate the interference of the NPs peak, a differential spectrum was obtained for different 50NPs–DOM (SI Figure S5). For the NPs-BC_DOM_, peaks at 3341, 1733, 1666, and (1411 and 1158) cm^–1^ represented the O—H, C=O, (C=O and C=C), and C—H stretching vibrations, respectively, which are the characteristic absorption peaks of BC.^56^ In the NPs-WS_DOM_, 3405 cm^–1^ represented the O—H stretching vibration, 2931 cm^–1^ represented the C—H stretching vibration in methylene, and 1735 cm^–1^ represented the C=O stretching vibration in ester carbonyl, indicating the presence of lipid-like compounds.^57^ The absorption peak at 1655 cm^–1^ was assigned to the protein in the amide I band.^58^ Although the absorption peak of the protein in amide II was concealed by miscellaneous peaks, the EEM results indicate the presence of plant proteins derived from wheat (Figure 2e). Moreover, the wide absorption peak at 1118 cm^–1^ indicated the presence of polysaccharides.^59^ Although cellulose does not easily dissolve in water, WS_DOM_ possibly contained cellulose fragments and starch owing to the high cellulose content (38.72%) and soluble starch (0.071%) in WS (SI Table S5). For NPs-SM_DOM_, 1654 cm^–1^ represented the protein absorption peak^60^ and 1009 cm^–1^ represented the C—O—C stretching vibration of the undigested cellulose^61^ in organic fertilizers. In general, changes were not observed in the absorption peaks of each functional group of the NPs. This indicates that no chemical reaction occurred between the NPs and different DOM. However, DOM can be physically adsorbed on NPs,^62,63^ and different organic components, including proteins, lipids, polysaccharides, and cellulose, can interact with NPs.

### Effect of DOM on Nanoplastics Transport

**Figure 3:**
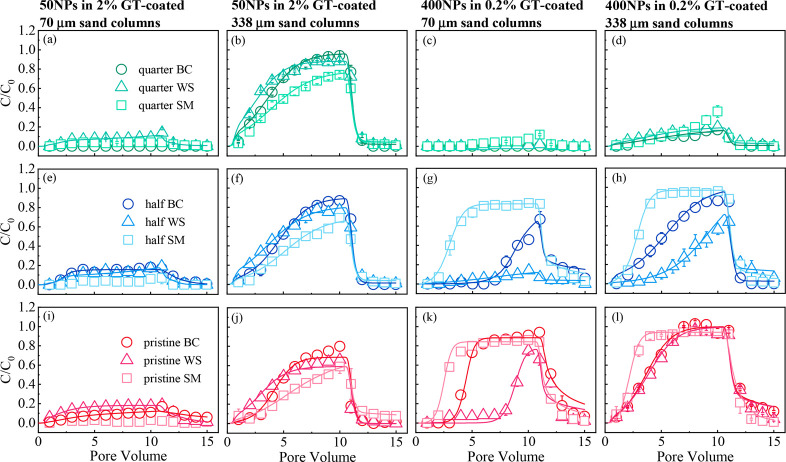
Breakthrough curves of 50NPs (a,b,e,f,i,j) and 400NPs (c,d,g,h,k,l) cotransport with different DOM with different concentrations in the 0.2 (a,b,e,f,i,j) and 2% (c,d,g,h,k,l) GT-coated 70 μm (a,c,e,g,i,k) and 338 μm (b,d,f,h,j, l) sand columns at an influent pH of 6.0. Symbols indicate observed data and solid lines indicate simulation fitting. Recovery rates of <5% in the effluent did not fit.

Cotransport of different-sized NPs and DOM was investigated in specific GT-coated sand columns (2% for 50NPs and 0.2% for 400NPs) and their BTCs and simulation results are shown in Figure 3 and SI Table S7, respectively. Regardless of the size of the sand and the GT contents, the transport of individual 50NPs was fast (Figure 1); however, the BTC height of NPs cotransported with DOM was significantly reduced. In the fine-sand columns (Figure 3a,e,i, the recovery rates of 50NPs in the effluent were 0%–18.5% (SI Table S7). Although the 50NPs transported at a certain rate in the coarse sand columns, their recovery rates decreased by 12.3%–48.5% compared to those of the NPs without DOM (Figure 3b,f,j and SI Table S7). The *k*_1a_ and *k*_2a_ values decreased and increased, respectively (SI Table S7), indicating that the reversible retention of 50NPs decreased, while their irreversible retention increased. The *k*_2a_ values in fine sand columns (1.957–2.910 min^–1^) were higher than those in coarse sand columns (0.025–0.372 min^–1^), consistent with the results observed for the transport characteristics.

**Figure 4:**
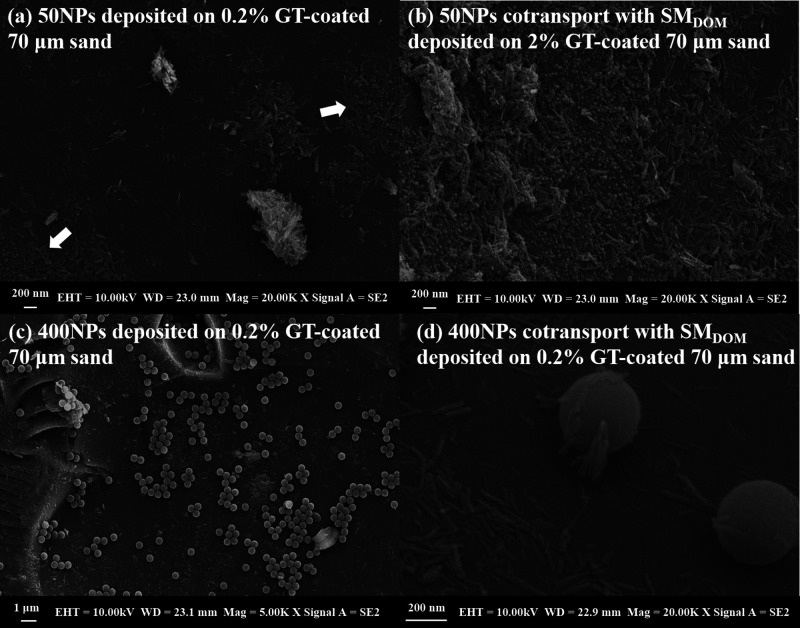
SEM images of 50NPs (a,b) and 400NPs (c,d) deposited on GT-coated sand from the 0–2.5 cm column layer.

A stable coexistence was observed for different DOM and 50NPs in the suspension (SI Figures S7 and S8a,b), and similar observations were made by a previous study.^64^ Therefore, the deposition of 50NPs could not be attributed to heteroaggregation with DOM. The DOM in the column exhibited three forms: GT adsorbed form, NPs bound form, and individual form. As the DOM gradually adsorbed on the GT during cotransport (Figure 4), the zeta potential of 2% GT-coated sand at the inlet (0–2.5 cm) changed from positive to negative (SI Table S3). For SM_DOM_ with high TOC concentrations, as well as, moderate zeta potential and HIX, the potential of the 2% GT-coated sand in the entire sand column was negative after adsorption (SI Table S3). This transition increased the electrostatic repulsion between the 50NPs and sand. The DLVO interaction energy between 50NPs and the sand after NPs cotransport with DOM showed that the primary energy barrier appeared at some positions, particularly in the columns eluted with SM_DOM_ (SI Figure S9a–f). Although the favorable chemical conditions of the 50NPs transport gradually improved and part of the deposition sites were occupied by DOM during the experiments, 50NPs transport was inhibited. The codeposition of DOM and 50NPs was attributed to the increased agglomeration of NPs by bridging and neutralization promoted by DOM and Fe minerals.^20,35^

Although the DOM concentrations in the fine sand columns varied between 0.37 and 31.4 mg L^–1^, the recovery rates of 50NPs were <18.5% (Figure 3a,e,i and SI Table S7), indicating that even a small amount of DOM caused the deposition of 50NPs. Small pores increased the contact opportunities among DOM, 50NPs, and GT, we therefore propose that the retention of 50NPs is due to their interaction.^65^ In coarse sand columns, the recovery rates of 50NPs decreased (from 54.5%–74.6% to 38.4%–47.0%) with increasing DOM concentrations (Figure 3b,f,j and SI Table S7), suggesting that a high DOM concentration enhanced 50NPs retention. The DOM for managing 50NPs deposition was mainly in GT adsorbed form and NPs bound form. Owing to the large pores available for 50NPs and individual DOM transport in coarse sand columns, the increase in deposition was low. For different sources of DOM, based on the environmental release capacity, SM_DOM_ exhibited the most evident retarding effect on 50NPs transport. Although the DOM concentration released by BC was the lowest, the retardation effect of BC_DOM_ with a high HIX (3.48) on the transport of 50NPs was comparable to that of WS_DOM_ with a low HIX (0.34), as WS_DOM_ were released in large quantities by wheat straw. This suggests that HA played a crucial role in 50NPs deposition. Additionally, the compositions of WS_DOM_ and SM_DOM_ were more complex than that of BC_DOM_, and a certain type of DOM might bind strongly to the NPs, thereby having a significant impact on their deposition.

The transport characteristics of 400NPs were the opposite to those of 50NPs, as DOM facilitated 400NPs transport (Figure 3). Similarly, a previous study reported that HA may promote the transport of approximately 500 nm NPs in manganese oxide-coated sand.^53^ In the present study, the promotion effect of a quarter of the DOM concentration on 400NPs transport was limited, particularly in the fine sand columns (Figure 3c,d). The promotion of 400NPs transport increased with increasing DOM concentration (Figure 3c,d,g,h,k,l), as confirmed by the increase in the recovery rates and decrease in the *k*_2a_ values (SI Table S7). Negatively charged functional groups (e.g., −COOH and −OH) were present on the DOM surface, which likely modified the surface charge of both the NPs and the GT-coated sand. Owing to the low GT content (0.2%), DOM significantly changed the zeta potential of the GT-coated sand (SI Tables S2 and S3). Therefore, the DLVO interaction energy between 400NPs and sand after cotransport also changed significantly. The interaction between 400NPs and the sand at different column positions presented an evident primary energy barrier (77.3–344.2 kT) (SI Figure S9g–l). These conditions may have caused the resumption of 400NPs transport. This indicates that GT adsorbed DOM mainly affected 400NPs transport by charge modification and competitive adsorption. The WS_DOM_ and SM_DOM_ did not contribute to the negative charge of 400NPs (SI Table S1); thus, the change in the surface charge properties of the GT-coated sand was considered as the key factor. The surface charge changed significantly in the coarse sand column (SI Table S3), and thus, 400NPs transported readily in the coarse sand column. Even in the 2% GT-coated fine sand columns, the adsorption of pristine- and half-SM_DOM_ during their transport fully changed the electrical properties of sand (SI Table S3); thus, it can be speculated that the transport of 400NPs would also be promoted under these conditions.

The changes in the distance and the range of the zeta potential of 0.2% GT-coated sand were small due to low HIX of WS_DOM_ (SI Table S3). Therefore, the promotion effect of WS_DOM_ on 400NPs transport was the lowest, while that of SM_DOM_ with moderate HIX and high concentration was the highest, even though SM_DOM_ caused low sedimentation of 400NPs (SI Figure S8c,d). BC_DOM_, with the highest HIX but low concentration, had a moderate promoting effect on 400NPs transport. In general, different DOM had different effects on 400NPs transport.

### Deposition of Nanoplastics

The depositional morphology of the NPs at the column inlet (0–2.5 cm) was analyzed using SEM (Figure 4). As the different DOM could not be distinguished, samples in the presence of SM_DOM_ were chosen as examples. The highlighted spheres on the rough surfaces in the SEM images indicated that NPs were deposited on the GT, regardless of the presence of DOM (Figure 4). Hence, it is reasonable to assume that adsorption occurred between Fe oxides and NPs,^13^ as well as between Fe and organic matter.^66^ Dense and single-layered 50NPs aggregates were bound to GT clusters in the presence of SM_DOM_ (Figure 4b and SI Figure S10a), while the deposition of 50NPs was sparse GT alone (Figure 4a), which is consistent with the results observed for the transport characteristics (Figure 3). For 400NPs with a high volume, 400NPs deposition as layers occurred in the presence of GT (Figure 4c and SI Figure S10b). The presence of DOM reduced their deposition, and their relationship was similar to that reported in a previous study, which reported that GT adsorbed onto plastic^13^ (Figure 4d and SI Figure S10b).

DOM entered at a high negative charge on the GT-coated sands (SI Table S3), resulting in high chemical heterogeneity. Moreover, the roughness of the sand can promote interactions that cause deposition, even under electrostatically unfavorable conditions.^67^ Nevertheless, chemical and physical heterogeneity contributed to changes in NPs mobility. Although the DLVO theory explained the transport of 400NPs, it could not clarify the deposition of 50NPs. The XPS results only identified the codeposition of NPs (C—C/C—H, 82.7–84.2%) and other DOM (C—O, O—C=O, 4.9–11.7%) (SI Figure S11), but it could not clarify the codeposition mechanism. The concentrations and types of DOM released by the agricultural organic inputs varied. In addition to the DOM concentration, HA plays an important role in 50NPs deposition and 400NPs transport. Moreover, other species of released DOM, such as lipids, proteins, and polysaccharides, from agricultural organic inputs possibly had various impacts on the fate of 50NPs and 400NPs, which requires further discussion.

### Interaction between Nanoplastics and Representative DOM

**Figure 5:**
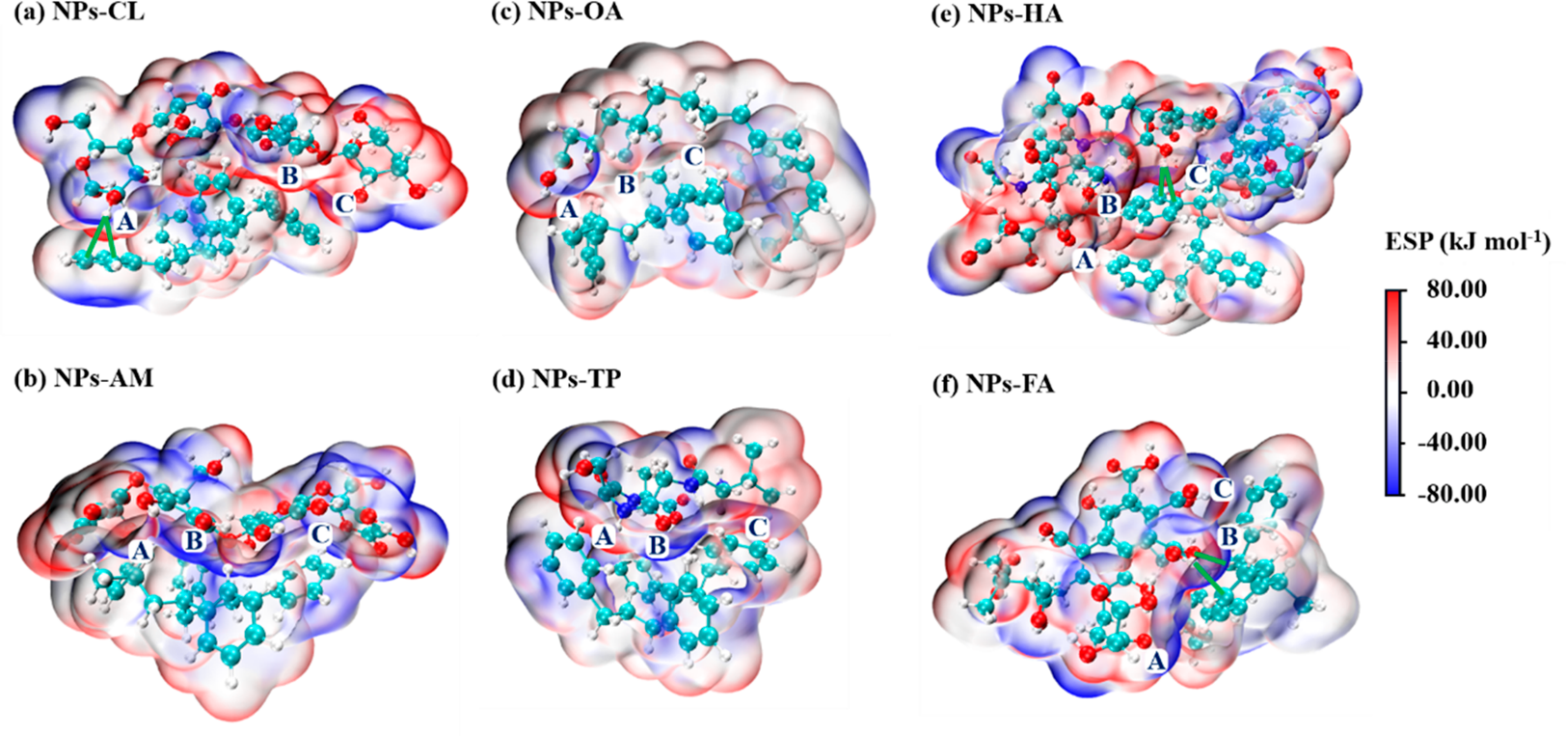
Cluster model of the structures of NPs and representative DOM: NPs-CL (a), NPs-AM (b), NPs-OA (c), NPs-TP (d), NPs-HA (e), and NPs-FA (f). The blue-green, white, red, and blue spheres represent C, O, H, and N, respectively, and the green lines represent hydrogen bonds. ESP indicates the electrostatic potential of the macromolecule, wherein red and blue are the positive and negative areas, respectively. Three key regions (A, B, and C) were identified for each cluster model. Their vertex coordinates and interpenetration distances are shown in SI Table S8. The binding energies between the representative NPs and DOM are shown in SI Table S9.

Although the interaction between DOM and NPs has been analyzed by FTIR spectra in this study and observed by SEM^35^ in previous studies, their microinterface mechanism requires further investigation. In this study, both the liquid phase and the GT-adsorbed DOM might have interacted with the NPs. Quantum chemical computations are widely used to calculate the interactions between NPs and organic matter.^68−71^ Various DOM species were derived from agricultural organic inputs. Although the quantum chemical calculations of the representative NPs–DOM systems cannot encompass all DOM species, they do provide a theoretical basis. In general, the NPs and representative DOM structures exhibited a surface penetration area, and van der Waals interaction (*I*_VDW_) and electrostatic interaction (*I*_ES_) held the molecules together. The benzene ring of NPs contained abundant π-electrons, which exhibited *I*_ES_ with a positively charged region of the representative DOM. Moreover, hydrogen bond interaction (*I*_HB_) enhanced the interaction between NPs and DOM (Figure 5). The interpenetration distances of key regions were in the range of 0.201–1.149 Å (SI Table S8).

In the NPs-CL system, with a binding energy of −178.29 kJ mol^–1^ (SI Table S9), the ESP on the surfaces of the two molecules overlapped in opposite directions (regions A, B, and C) (Figure 5a). In area A, *I*_HB_ was observed between the hydrogen atom of the hydroxyl group on the CL and the carbon atom of the benzene ring on the NPs, which further enhanced the interaction. Although *I*_VDW_ and *I*_ES_ were evident in the NPs-AM system, *I*_HB_ did not occur, (Figure 5b), causing a decrease in the binding energy to −158.61 kJ mol^–1^. (SI Table S9). In the NPs-OA system, only charge distribution was present near the carboxyl group of OA (region A) (Figure 5c). Although an OA chain surrounded the NPs and the interpenetration distance of the key areas was very small (0.168–0.359 Å) (SI Table S8), the binding energy of the system was low (−117.76 kJ mol^–1^) (SI Table S9) due to the absence of *I*_ES_. Previous studies suggested that lipids and NPs (polyethylene) dominantly interacted via *I*_VDW_,^68^ and that the ESP was low (−57.6 kJ mol^–1^).^71^ Therefore, lipids were considered to have a weak effect on NPs transport. In the NPs-TP system, regions A and C exhibited an overlap of positive and negative potentials (Figure 5d). The binding energy of this system was −121.81 kJ mol^–1^, which was higher than that of the NPs-OA system (SI Table S9).

In the NPs-HA and NPs-FA systems, *I*_BD_ occurred between the carbon atom of the benzene ring on NPs and either the hydrogen atom in the hydroxyl group on HA (region C) (Figure 5e) or the hydrogen and oxygen atoms in the carboxyl group on FA (region B) (Figure 5f). Moreover, the positive and negative potentials in the two systems overlapped. The binding energies of the NPs-HA and NPs-FA system were −169.92 and −166.08 kJ mol^–1^, respectively, which were close to that of the NPs-CL system. Although HA and FA had similar binding energies to NPs, on a large scale, HA with a chain-like structure may assume an intertwined conformation with NPs, which is more conducive to form electrostatic potential overlap on their surfaces and the hydrogen bonds. Moreover, intertwined conformation likely led to multipoint attachment between NPs and HA (1.020–1.113 Å) (SI Table S8). It is known that NPs are polarized when they are near the HA polar group, and the induced dipole and inherent dipole are attracted to one another, which generates an induced force. Therefore, inconsistent instantaneous positive and negative charge centers of gravity may be formed when NPs come close to the hydrophobic group of HA, generating transient dipoles and a dispersion force.^68,71^ A previous study reported that HA adsorption on MPs was higher than that of FA,^63^ which supported our hypothesis. Intertwined conformation might occur between the long-chain CL and NPs. A similar mechanism of bridging coagulation has been applied to MPs removal by chain-like structure protein amyloid fibrils,^73^ confirming the strong interaction between NPs and long-chain DOM.

### Mechanism of Cotransport of Nanoplastics and DOM

#### Particle Size Selectivity

According to the DLVO theory, NPs–DOM complexes are repelled by electrostatic interaction as approach the DOM-loaded GT-coated sand. The repulsion for 50NPs (0–27.0 kT) was much lower than that for 400NPs (77.3–344.2 kT) (SI Figure S9). This resulted in opportunities for 50NPs to get closer to the sand. A study reported that the DLVO model is inaccurate for short interparticle distances owing to a non-DLVO attractive interaction within a range of ∼3 nm.^75^ Furthermore, this non-DLVO attraction may essentially be invariant with salinity and is likely obtained mostly or entirely from specific charge–charge Coulomb interactions in the electric double layer.^74^ In that case, when the distance between 50NPs–DOM complex and GT-coated sand is less than 3 nm, DOM adsorption likely occurred on GT due to Coulomb interactions^74^ and ligand exchange.^66,75^ Subsequently, the formation of GT–DOM–50NPs complexes retarded 50NPs transport. For the transport of small NPs, non-DLVO attraction may be more significant than DLVO force. The small pore structure,^29,65^ surface roughness of GT-coated sand,^71,76,77^ and morphology of GT^78^ increased the probability of GT–DOM–50NPs bond formation, thereby providing favorable conditions for 50NPs deposition. Although quantum interactions occurred between DOM and 400NPs, it was difficult to form GT–DOM–400NPs complexes because 400NPs were repulsed from from GT-coated sand by electrostatic and steric repulsion by the DOM attached to nanoparticles and sand.^36^

#### Role of DOM

Various organic matter components were pyrolyzed during the preparation of BC, and BC_DOM_ components were relatively simple. As the HIX was high (3.48) for BC_DOM_ (Figure 2d), HA was considered as the main component. Although the HA concentration was low, it likely retarded the transport of 50NPs (Figure 3) because of the strong interaction between NPs and HA. The components of WS_DOM_ were complex and contained different DOM species. Although the HIX was low, WS_DOM_ was rich in CL fragments derived from the straw cell walls, which strongly interacted with the WS_DOM_ and NPs. Moreover, WS_DOM_ was readily deposited in the fine sand column with a high GT content (SI Figure S6 and Table S6), which facilitated its codeposition with 50NPs. Thus, WS_DOM_ had a retardation effect on 50NPs transport that was similar to that of BC_DOM_ (Figure 3). Furthermore, SM_DOM_ had a complex composition with a high concentration. This suggests that the moderate degree of humification and CL content are probably key characteristics of SM_DOM_, and HA and CL have strong interactions with 50NPs, resulting in the highest retardation effect on their transport (Figure 3). Lipids and proteins were also important components of WS_DOM_ and SM_DOM_; however, their representative substances had low interactions with NPs (66.0–71.7% of that between NPs and CL/HA) and therefore, may contribute little to deposition. Moreover, lipids, proteins, and starch have short-term impacts on NPs deposition as they are easily degraded. In contrast, the influences of CL, HA, and FA might have prolonged effects due to their stability in the soil. In the long term, the retention of microbiologically available WS_DOM_ and SM_DOM_ was conducive to biofilm formation, and thus, causing the NPs retardation by biochemical effects.^15^ Although the influent conditions were unified by 10-mM NaCl, the ions released during DOM extraction promoted the formation of GT–DOM–50NPs,^35^ especially in SM_DOM_.

## Environmental Implications

Farmland soil may represent one of the largest environmental sinks for plastics pollution.^79^ Our findings highlight the significant environmental implications of agricultural organic inputs on farmland soils containing NPs. After the application of these inputs in agricultural production, the DOM concentrations significantly increased in the soil pore water. The addition of 5% biochar increased the soil DOM by >5 mg L^–1^, while DOM leaching significantly increased.^80^ Additionally, 13–60% DOM was derived from the straw that was returned to agricultural soil,^81^ and organic fertilization doubled soil DOM concentrations.^82^ In this case, abundant DOM in agricultural soils might cause the downward transport of large NPs, while small NPs were retained in the tillage layer. The application of organic fertilizers may further promote NPs size differentiation during transport. Furthermore, NPs retention may have direct or indirect knock-on effects on plants and crop yields^18^ and can accumulate in the roots and migrate to the shoots.^83^ Because NPs are toxic to most life forms and can transport metallic and organic pollutants,^84^ their accumulation in the tillage layer increases the risk of NPs transmission from the food chain to human, which requires further attention.

## Supporting Information Available

Goethite preparation, The calibration curves of molecular weights of DOM and humification index calculation, The calibration curves of concentrations of nanoplastics and nanoplastics with DOM, Zeta potential of experimental material, Nanoparticles transport models, XDLVO theory, Typical DOM selection reason and their molecular formula, Calculation of binding energy, Fitted parameters of nanoplastics transport in the GT coated sand columns, DLVO interaction energy between nanoplastics and (GT coated) sand, The contents of starch, hemicellulose, cellulose, and lignin in the agricultural organic inputs, FTIR differential spectra analysis, Transport of different DOM, Fitted parameters of DOM transport in the GT coated sand columns, Fitted parameters of nanoplastics cotransport with DOM in the GT coated sand columns, Stability of DOM and its influence on the stability of nanoplastics, DLVO interaction energy between nanoplastics and sand before and after cotransport with DOM in the GT coated sand columns, Deposition of nanoplastics, XPS results of nanoplastics and different DOM codeposited on GT coated sand, Result parameters of quantum chemical computation (PDF)
