# Supplementary material for: Effect of Agricultural Organic Inputs on Nanoplastics Transport in Saturated Goethite-Coated Porous Media: Particle Size Selectivity and Role of Dissolved Organic Matter
Source: Environ Sci Technol. 2022 Feb 28;56(6):3524–34. doi: 10.1021/acs.est.1c07574 (PMC8928475; doi:10.1021/acs.est.1c07574)
Supplement: Supplementary file 1 — es1c07574_si_001.pdf [file es1c07574_si_001.pdf]

Supplementary Material

Effect of agricultural organic inputs on nanoplastics transport in saturated goethite-coated porous media：Particle size selectivity and role of dissolved organic matter

Jie Ma [a, b], Yan Qiu [c], Junying Zhao [c], Xiaoxue Ouyang [a, b], Yujie Zhao [a, b], Liping Weng [a, b, d *], MD Yasir Arafat [a, b], Yali Chen [a, b], Zongling Ren [e], Yongtao Li [f, e]

[a] Key Laboratory for Environmental Factors Control of Agro-Product Quality Safety, Ministry of Agriculture and Rural Affairs, Tianjin, 300191, China

[b] Agro-Environmental Protection Institute, Ministry of Agriculture and Rural Affairs, Tianjin 300191, China

[c] School of Environmental Science and Safety Engineering, Tianjin University of Technology, Tianjin, 300384, China

[d] Department of Soil Quality, Wageningen University, Wageningen, The Netherlands

[e] College of Resource and Environmental Engineering, Jiangxi University of Science and Technology, Ganzhou Jiangxi, 341000, China

[f] College of Natural Resources and Environment, South China Agricultural University, Guangzhou, 510642, China

*Corresponding Author

Email address: majie@caas.cn (Jie Ma), wengliping@caas.cn (Liping Weng).

List of contents

S1. Goethite preparation

S2. The calibration curves of molecular weights of DOM and humification index calculation

S3. The calibration curves of concentrations of nanoplastics and nanoplastics with DOM

S4. Zeta potential of experimental material

S5. Nanoparticles transport models

S6. XDLVO theory

S7. Typical DOM selection reason and their molecular formula

S8. Calculation of binding energy

S9. Fitted parameters of nanoplastics transport in the GT coated sand columns

S10. DLVO interaction energy between nanoplastics and (GT coated) sand

S11. The contents of starch, hemicellulose, cellulose, and lignin in the agricultural organic inputs

S12. FTIR differential spectra analysis

S13. Transport of different DOM

S14. Fitted parameters of DOM transport in the GT coated sand columns

S15. Fitted parameters of nanoplastics co-transport with DOM in the GT coated sand columns

S16. Stability of DOM and its influence on the stability of nanoplastics

S17. DLVO interaction energy between nanoplastics and sand before and after co-

transport with DOM in the GT coated sand columns

S19. Deposition of nanoplastics

S20. XPS results of nanoplastics and different DOM co-deposited on GT coated sand

S21. Result parameters of quantum chemical computation

S1. Goethite preparation

Goethite was prepared with adding 2.5 M NaOH at a speed of 10 ml min^{-1} to 5 L 0.5 M $Fe(NO_3)_3$ solution. Keep on mixing the suspension during the addition. And put a pH-electrode in the above suspension to monitor the pH and stop adding NaOH when pH = 12. Then put the suspension in an oven at 60 °C for 4 days to let $Fe(OH)_3$ age. At last, decant the clear solution on the top and dialyze $Fe(OH)_3$ paste until the EC < 10 μS.

S2. The calibration curves of molecular weights of DOM and humification index calculation

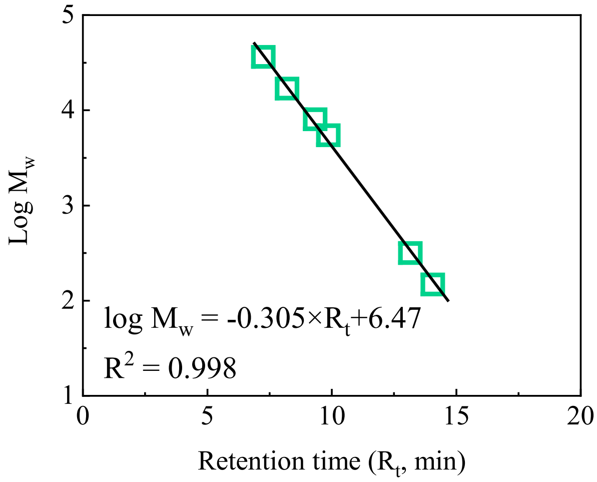

Figure S1. The calibration curves of molecular weights of DOM between retention time and standard substances.

According to the soluble organic components detected in the fluorescence spectral distribution, excitation/emission (E_x/E_m) wavelength regions can be considered as humic acid-like areas to demonstrate the humic characteristics of these components [1]. The humification index (HIX) was calculated as the ratio of the peak integrated area of emission wavelengths ranging from 300 to 345 nm to that of emission wavelengths ranging from 435 to 480 nm, under a 255-nm excitation wavelength [2].

S3. The calibration curves of concentrations of nanoplastics and nanoplastics with DOM

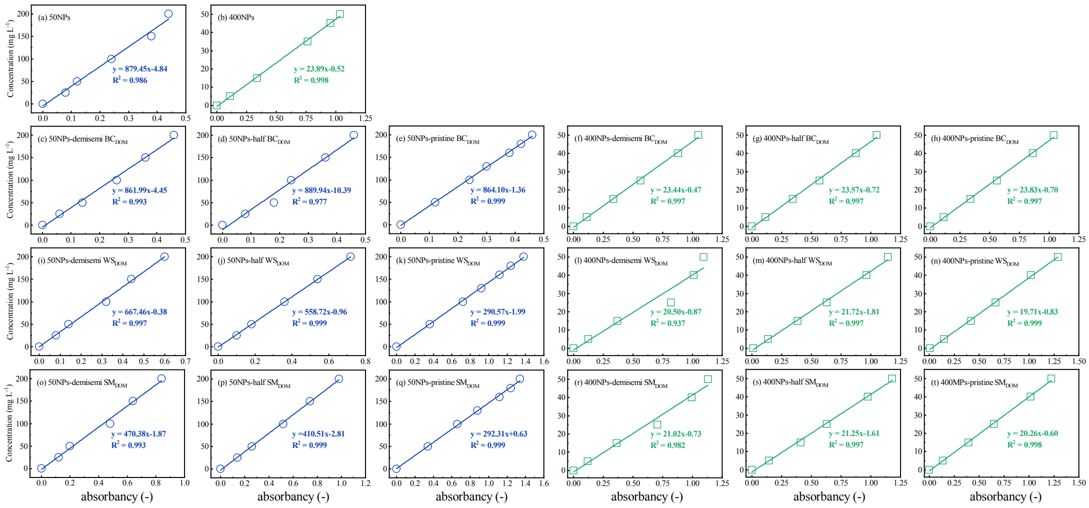

Figure S2. The calibration curves of 50NPs (a), 400NPs (b), and NPs-DOM suspension (c-t) concentrations between absorbency and standards at pH 6.0.

S4. Zeta potential of experimental material

Table S1 Zeta potential of NPs and NPs-DOM

DOM	Zeta potential (mV)	
	50NPs	400NPs
-	-39.8 ± 1.2	-40.6 ± 0.7
pristine BC_{DOM}	-57.2 ± 2.1	-58.1 ± 1.9
pristine WS_{DOM}	-30.4 ± 1.5	-28.9 ± 1.3
pristine SM_{DOM}	-35.0 ± 0.8	-33.2 ± 1.1
half BC_{DOM}	-54.0 ± 0.6	-55.3 ± 0.5
half WS_{DOM}	-32.4 ± 1.8	-31.7 ± 1.1
half SM_{DOM}	-35.9 ± 1.4	-34.6 ± 0.5
quarter BC_{DOM}	-53.6 ± 0.9	-54.0 ± 0.2
quarter WS_{DOM}	-34.3 ± 1.7	-33.2 ± 0.6
quarter SM_{DOM}	-36.6 ± 0.7	-36.1 ± 1.0

Table S2 Zeta potential of quartz and GT coated quartz.

Collector	Quartz	0.2% GT-Quartz	0.5% GT-Quartz	2% GT-Quartz
Zeta potential (mV)	-29.2 ± 1.6	19.8 ± 0.6	26.1 ± 0.9	30.2 ± 1.1

Table S3 Zeta potential of GT-coated sand (0-2.5, 2.5-5, 5-7.5, and 7.5-10 cm) after co-transport experiment of NPs and DOM.

Column	Zeta potential (mV)			
	0-2.5 cm	2.5-5 cm	5-7.5 cm	7.5-10 cm
2% GT-70μm Quartz pristine BC_{DOM}	-16.8 ± 5.1	-12.4± 1.1	-3.9 ± 2.1	8.7 ± 3.1
2% GT-70μm Quartz pristine WS_{DOM}	-10.7 ± 1.4	5.0 ± 2.0	16.9 ± 2.2	24.6 ± 1.6
2% GT-70μm Quartz pristine SM_{DOM}	-27.5 ± 1.8	-21.2 ± 0.6	-17.6 ± 2.0	-15.5 ± 0.5
2% GT-338μm Quartz pristine BC_{DOM}	-15.7 ± 2.6	-11.1 ± 0.8	-6.6 ± 1.0	-3.4 ± 1.3
2% GT-338μm Quartz pristine WS_{DOM}	-6.4 ± 2.8	9.2 ± 2.1	20.5 ± 0.5	25.3 ± 0.1
2% GT-338μm Quartz pristine SM_{DOM}	-24.4 ± 1.3	-22.2 ± 1.6	-19.5 ± 1.8	-18.7 ± 1.0
0.2% GT-70 μm Quartz pristine BC_{DOM}	-28.7 ± 0.6	-28.2 ± 0.2	-27.3 ± 1.0	-22.3 ± 1.4
0.2% GT-70 μm Quartz pristine WS_{DOM}	-26.4 ± 0.9	-23.6 ± 0.5	-16.0 ± 1.7	-14.3 ± 2.2
0.2% GT-70 μm Quartz pristine SM_{DOM}	-29.2 ± 0.1	-29.0 ± 0.5	-28.4 ± 0.4	-27.7 ± 0.8
0.2% GT-338 μm Quartz pristine BC_{DOM}	-29.8 ± 0.1	-27.2 ± 0.3	-26.2 ± 0.2	-24.3 ± 0.6
0.2% GT-338 μm Quartz pristine WS_{DOM}	-25.4 ± 0.4	-22.0 ± 0.7	-20.3 ± 0.9	-15.6 ± 1.2
0.2% GT-338 μm Quartz pristine SM_{DOM}	-29.0 ± 0.5	-27.0 ± 0.1	-26.4 ± 1.3	-25.8 ± 0.7
2% GT-70μm Quartz half BC_{DOM}	16.2 ± 5.1	21.5 ± 2.4	23.9 ± 0.9	28.7 ± 2.2
2% GT-70μm Quartz half WS_{DOM}	22.6 ± 0.8	25 ± 1.4	26.3 ± 3.4	28.6 ± 2.5
2% GT-70μm Quartz half SM_{DOM}	-17.4 ± 1.7	-9.2 ± 4.3	-7.6 ± 2.7	-5.5 ± 1.2
2% GT-338μm Quartz half BC_{DOM}	-6.8 ± 3.1	5.4 ± 1.3	17.5 ± 1.9	23.4 ± 2.0
2% GT-338μm Quartz half WS_{DOM}	15.3 ± 3.6	19.7 ± 2.2	24.0 ± 1.4	27.1 ± 1.8
2% GT-338μm Quartz half SM_{DOM}	-20.2 ± 0.2	-19.1 ± 0.6	-18.3 ± 1.3	-17.9 ± 1.1
0.2% GT-70 μm Quartz half BC_{DOM}	-18.4 ± 1.8	-14.7 ± 0.4	-11.6 ± 1.3	-7.1 ± 2.1
0.2% GT-70 μm Quartz half WS_{DOM}	-14.5 ± 6.2	-7.7 ± 2.3	-5.0 ± 3.4	4.0 ± 3.4
0.2% GT-70 μm Quartz half SM_{DOM}	-24.4 ± 2.0	-23.7 ± 1.4	-22.3 ± 1.0	-21.0 ± 1.8
0.2% GT-338 μm Quartz half BC_{DOM}	-20.5 ± 0.5	-19.8 ± 1.1	-19.2 ± 0.7	-17.3 ± 0.4
0.2% GT-338 μm Quartz half WS_{DOM}	-21.4 ± 1.2	-14.6 ± 2.6	-9.6 ± 1.7	-5.3 ± 2.9
0.2% GT-338 μm Quartz half SM_{DOM}	-24.7 ± 1.5	-23.3 ± 0.2	-21.8 ± 1.8	-20.8 ± 1.5
2% GT-70μm Quartz quarter BC_{DOM}	-4.2 ± 2.6	16.1 ± 2.4	27.5 ± 0.5	29.4 ± 1.9
2% GT-70μm Quartz quarter WS_{DOM}	25.6 ± 0.4	28.7 ± 0.5	30.2 ± 1.1	30.3 ± 0.2
2% GT-70μm Quartz quarter SM_{DOM}	-16.6 ± 1.8	1.2 ± 5.1	22.6 ± 3.2	29.8 ± 1.3
2% GT-338μm Quartz quarter BC_{DOM}	2.7 ± 4.2	9.8 ± 4.0	28.5 ± 1.3	29.6 ± 0.6
2% GT-338μm Quartz quarter WS_{DOM}	21.5 ± 2.1	27.4 ± 2.0	28.8 ± 1.6	30.2 ± 0.3
2% GT-338μm Quartz quarter SM_{DOM}	-13.9 ± 4.2	-7.1 ± 3.3	-2.3 ± 3.8	8.5 ± 3.0
0.2% GT-70 μm Quartz quarter BC_{DOM}	-11.4 ± 1.8	-5.1 ± 2.4	4.6 ± 3.3	14.1 ± 2.1
0.2% GT-70 μm Quartz quarter WS_{DOM}	-11.5 ± 6.2	-3.7 ± 2.3	7.0 ± 4.4	15.5 ± 2.1
0.2% GT-70 μm Quartz quarter SM_{DOM}	-21.2 ± 1.9	-14.1 ± 2.2	-5.7 ± 3.8	7.0 ± 2.4
0.2% GT-338 μm Quartz quarter BC_{DOM}	-12.3 ± 0.8	-6.8 ± 1.4	3.1 ± 1.7	12.5 ± 2.1
0.2% GT-338 μm Quartz quarter WS_{DOM}	-11.7 ± 2.6	-4.0 ± 3.5	9.2 ± 3.2	16.7 ± 2.2
0.2% GT-338 μm Quartz quarter SM_{DOM}	-20.2 ± 1.9	-13.5 ± 2.7	-7.4 ± 1.7	5.9 ± 3.3

S5. Nanoparticles transport models

The convection diffusion equation (CDE) with two kinetic retention sites was employed to describe the nanoparticle transport and retention in the column experiments as equation (1) [3, 4].

$$\frac{\partial C}{\partial t} = -\frac{v\partial C}{\theta\partial x} + D\frac{\partial^2 C}{\partial x^2} - \frac{\rho\partial S_1}{\theta\ \partial t} - \frac{\rho\partial S_2}{\theta\ \partial t} \qquad (1)$$

θ ($cm^3 \cdot cm^{-3}$) is the volumetric water content; D is the dispersion coefficient ($m^2 \cdot s^{-1}$); ρ ($g \cdot m^{-3}$) is the column dry bulk density; x (cm) is the spatial coordinate; ν ($cm \cdot min^{-1}$) is the Darcy's velocity; and S_1 ($g \cdot g^{-1}$) and S_2 ($g \cdot g^{-1}$) are nanoparticle concentrations deposited in Site1 and Site2, respectively.

The Site1, first kinetic site, on which the retention of the nanoparticle is assumed to be reversible, whereas Site2, the second kinetic site, on which the retention is assumed to be irreversible, as described by the depth-dependent retention. S_1 on Site1 and S_2 on Site2 are given in equations (2) and (3), respectively.

$$\frac{\rho\partial S_1}{\theta\ \partial t} = k_{1a}C - \frac{\rho}{\theta}k_{1d}S_1 \qquad (2)$$

$$\frac{\rho\partial S_2}{\theta\ \partial t} = \psi_t k_{2a}C \qquad (3)$$

k_{1a} (min^{-1}) and k_{2a} (min^{-1}) are first-order retention coefficients on Site1 and Site2, respectively; k_{1d} (min^{-1}) is the first-order detachment coefficient; ψ_t (dimensionless) is the nanoparticle attachment function to account for the depth-dependent behavior of particle attachment expressed by equations (4):

$$\psi_t = \left(\frac{d_c + x - x_0}{d_c}\right)^{-\beta} \qquad (4)$$

d_c is the median diameter of the sand grains (cm); x_0 is the coordinate of the location where the straining process starts; and β (dimensionless) is an empirical

variable that controls the shape of the retention profile, using an optimal value of 0.432 for different sized spherical nanoparticle and sand grains in which significant depth-dependency (hyperexponential retention profiles) occurred [4]. Three parameters, including k_{1a}, k_{2a}, and k_{1d}, were fitted.

S6. DLVO theory

The representative Derjaguin-Landau-Verwey-Overbeek (DLVO) theory was used to qualitatively understand the NPs transport and retention in water-saturated sands columns through calculating the total particle-sand interaction energy as the sum of Lifshitz-van der Waals (LW) and electrical double layer (EDL) interactions [5, 6]. Ionic strength stays constant at 0.1 mM NaCl. The equation of the LW interaction energy (E_{LW}) is given as follows [7, 8]:

$$E_{LW} = -\frac{A_{132} d_p}{12h}\left[1 + \frac{14h}{\lambda}\right]^{-1} \quad (5)$$

d_p is the diameter of nanoparticle; h is the separation distance between the nanoparticle and sand surface; λ is the characteristic wavelength of interaction and was defined as 100 nm; A_{132} is the Hamaker constant of particle-water-sand, which can be expressed by equation (6):

$$A_{132} = \left(\sqrt{A_{11}} - \sqrt{A_{33}}\right)\left(\sqrt{A_{22}} - \sqrt{A_{33}}\right) \quad (6)$$

A_{11} is the Hamaker constant for NPs (6.60×10^{-20} J) [9]; A_{22} is the Hamaker constant for quartz sand (8.86×10^{-20} J) [10]; A_{33} is the Hamaker constant for water (3.7×10^{-20} J) [9].

The equation of EDL interaction energy (E_{EDL}) is given as follows [11, 12]:

$$E_{EDL} = 0.5\pi\varepsilon_0\varepsilon_r d_p\left\{2\psi_p\psi_c \ln\left[\frac{1 + \exp(-\kappa h)}{1 - \exp(-\kappa h)}\right] + (\psi_p^2 + \psi_c^2)\ln[1 - \exp(-2\kappa h)]\right\} \quad (7)$$

ε_0 is the dielectric permittivity of vacuum (8.854×10^{-12} F·m^{-1}); ε_r is the relative dielectric permittivity of water (78.5); ψ_p and ψ_c are the zeta potentials of NPs and GT-coated sand, respectively; κ (m^{-1}) is the Debye-Hüchel parameter, which is expressed by equation (8);

$$\kappa = 3.28 \times 10^9 (I)^{1/2} \qquad (8)$$

I is ionic strength.

S7. Typical DOM selection reason and their molecular formula

A part of the polysaccharides in WS_{DOM} and SM_{DOM} may be directly or indirectly derived from the cellulose (CL) in plant cell walls; thus, CL was selected to represent polysaccharides. Amylose (AM) was also selected as a common polysaccharide. Both plant and animal lipids mostly comprise long chains with an ester carbonyl group; thus, oleic acid (OA) represented lipid-like compounds. Moreover, a tetrapeptide (TP, valine-glycine-serine-alanine) was chosen to represent proteins. Furthermore, HA and fulvic acid (FA) were considered as typical DOM. Two hundred original configurations were generated, and each configuration was then optimized based on Parameterized Model number 6. The first thirty configurations with the lowest energies were further optimized based on the all-electron density functional theory (DFT) at B3LYP/3-21G(d) level using Gaussian 16[13]. The optimized configuration with the lowest energy was then further optimized at B3LYP/6-31G(d) level. Finally, the single-point energy was calculated at the B3LYP/6-311G(d) level. Grimme's D3BJ dispersion was used to describe the inter-molecular interactions.

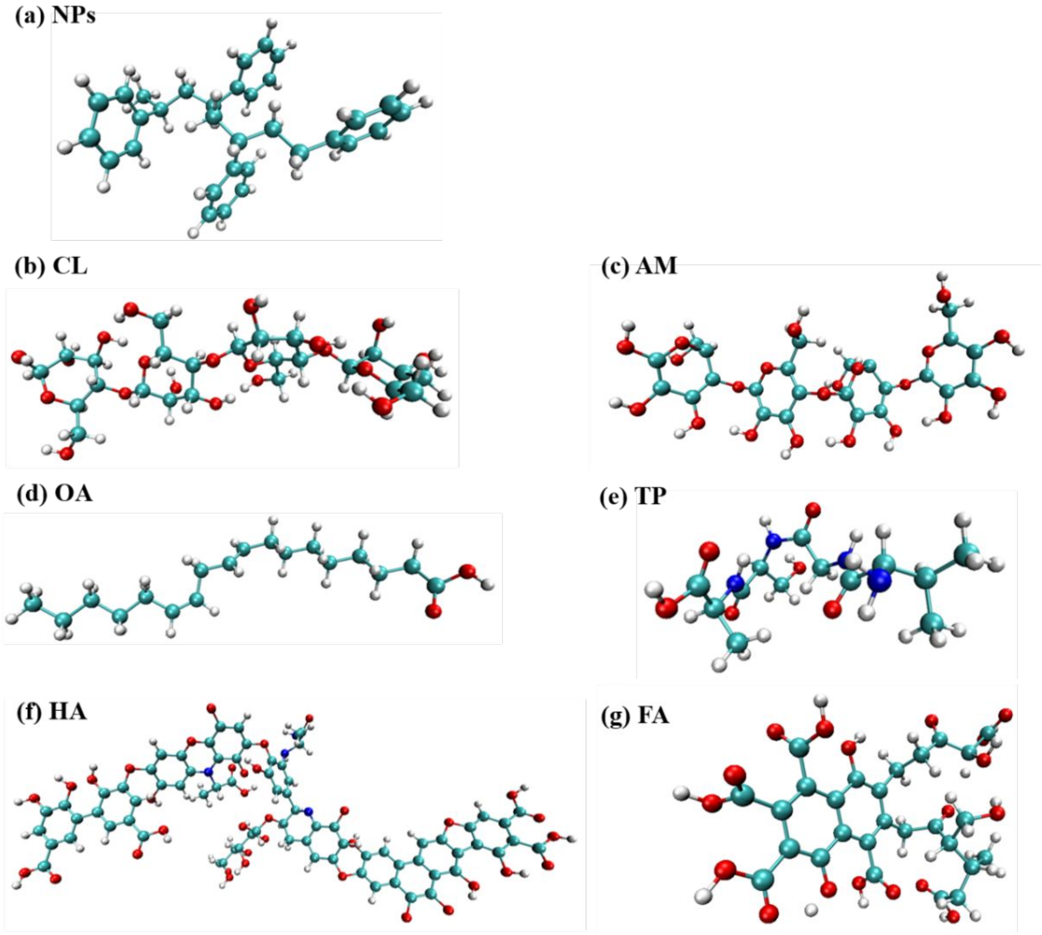

Figure S3. Molecular formula of typical DOM, molecular structure of HA and FA cited from Ouni, et al. [14]. The blue-green, white, red, and blue spheres represent C, O, H, and N, respectively.

S8. Calculation of binding energy

The equation of binding energy between different species NPs and DOM is given as follows:

$$\text{Binding energy} = E_{complex} - (E_{fragment1} + E_{fragment2}) \qquad (9)$$

Where $E_{complex}$ represents the energy of a complex composed of two molecules, and $E_{Fragment1}$ and $E_{Fragment2}$ represent the energy of a single molecule corresponding to different systems.

S9. Fitted parameters of nanoplastics transport in the GT coated sand columns

Table S4. Fitted parameters of NPs transport in different ratio GT coated 70 μm and 338 μm sand columns at pH 6.0.

NPs	Column	k_{1a} [a] (min^{-1})	k_{1d} [b] (min^{-1})	k_{2a} [c] (min^{-1})	k_{1d}/k_{1a}	R^2 [d]	Recovery [e] (%)
50NPs	70 μm Quartz	0.20 ± 0.13	0.617 ± 0.396	0.032 ± 0.010	3.02±0.03	0.995 ± 0.002	98.8 ± 0.9
	0.2% GT-70 μm Quartz	1.22 ± 0.35	0.008 ± 0.002	0.000 ± 0.000	0.006±0.000	0.988 ± 0.001	88.0 ± 1.3
	0.5% GT-70 μm Quartz	1.59 ± 0.13	0.001 ± 0.000	0.013 ± 0.007	0.000±0.000	0.998 ± 0.001	75.8 ± 2.5
	2% GT-70 μm Quartz	1.16 ± 0.02	0.000 ± 0.000	0.000 ± 0.000	0.000±0.000	0.996 ± 0.001	59.1 ± 2.3
	338 μm Quartz	0.03 ± 0.00	0.324 ± 0.196	0.000 ± 0.000	13.17±9.156	0.959 ± 0.007	106.6 ± 0.2
	0.2% GT-338 μm Quartz	0.45 ± 0.19	0.003 ± 0.001	0.000 ± 0.000	0.007±0.000	0.99 6± 0.003	92.9 ± 2.2
	0.5% GT-338 μm Quartz	0.34 ± 0.07	0.024 ± 0.001	0.000 ± 0.000	0.074±0.012	0.969 ± 0.002	94.5 ± 2.7
	2% GT-338 μm Quartz	0.38 ± 0.03	0.003 ± 0.002	0.014± 0.004	0.008±0.005	0.985 ± 0.006	86.9 ± 1.4
400NPs	70 μm Quartz	2.17 ± 2.07	4.33 ± 4.11	0.114 ± 0.005	2.07±0.08	0.993 ± 0.006	91.9 ± 1.4
	0.2% GT-70 μm Quartz	-	-	-	-	-	0.1 ± 0.0
	338 μm Quartz	3.41 ± 0.20	7.93 ± 0.46	0.031 ± 0.003	2.32±0.00	0.999 ± 0.000	94.9 ± 0.5
	0.2% GT-338 μm Quartz	0.62 ± 0.18	0.000 ± 0.000	1.112 ± 0.581	0.000±0.000	0.963 ± 0.022	6.3 ± 1.2

[a] The first-order retention coefficient on Site1.

[b] The first-order detachment coefficient on Site1.

[c] The first-order retention coefficient on Site2.

[d] Squared Pearson's correlation coefficient.

[e] Recovery of NPs in the effluent.

S10. DLVO interaction energy between nanoplastics and (GT-coated) sand

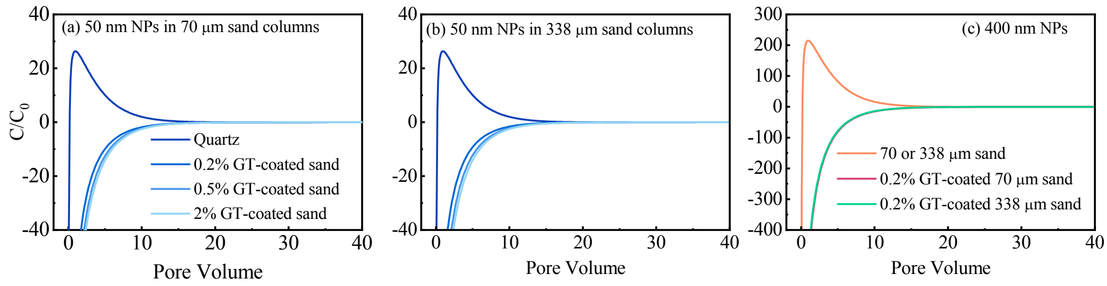

Figure S4. DLVO interaction energy (E_{TOT}) between NPs and (GT-coated) sand. The E_{TOT} is expressed in kT, where k is the Boltzmann constant and T is the absolute temperature in Kelvin.

S11. The contents of starch, hemicellulose, cellulose, and lignin in the agricultural organic inputs

The contents of starch, hemicellulose, cellulose, and lignin in BC, SW, and SM were determined using an enzymatic method [15, 16].

Table S5. The contents of starch, hemicellulose, cellulose, and lignin in the BC, SW, and SM

Agricultural organic inputs	starch (%)	hemicellulose (%)	cellulose (%)	lignin (%)
BC	0.000 ± 0.000	0.00 ± 0.00	0.75 ± 0.08	2.75 ± 0.08
WS	0.071 ± 0.014	28.14 ± 0.19	38.72 ± 0.14	7.17 ± 0.20
SM	0.057 ± 0.009	9.54 ± 0.44	10.05 ± 0.16	7.39 ± 0.36

S12. FTIR characteristics of nanoplastics and FTIR differential spectra analysis

The series absorption peaks at 3085, 3062, and 3025 cm^{-1} were attributed to the C-H stretching vibration of the benzene ring, and the series absorption peaks at 2924 and 2852 cm^{-1} were assigned to the C-H stretching vibration of methylene. The stair-stepping peaks at 1604 cm^{-1}, 1494 cm^{-1}, and 1451 cm^{-1} were related to the C=C stretching vibration of the benzene ring, and the absorption peaks at 756 cm^{-1} and 700 cm^{-1} were assigned to the C-H bending vibration of the benzene ring.

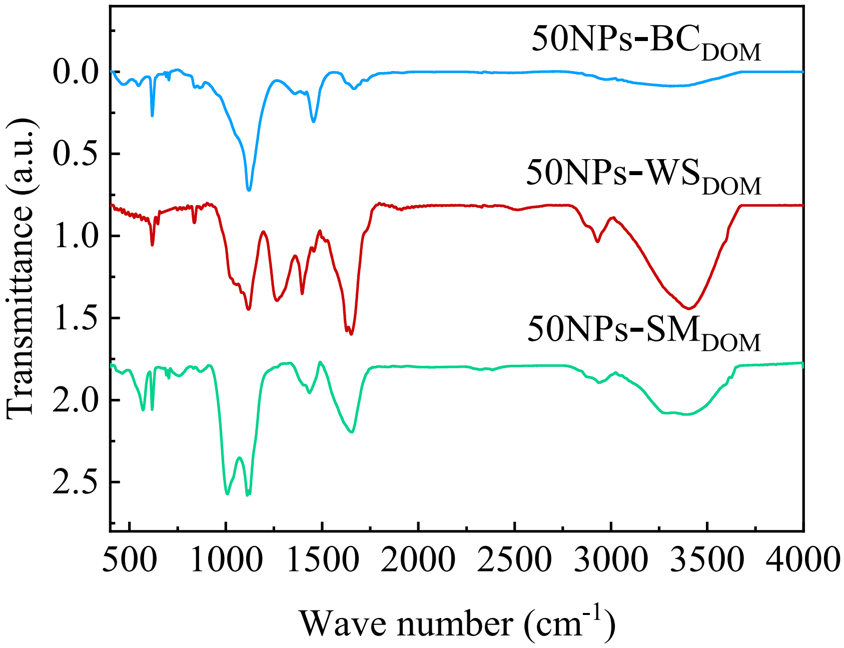

Figure S5. FTIR differential spectra analysis between different 50NPs-DOM and 50NPs

S13. Transport of different DOM

For individual DOM transport, particularly in 2% GT-coated 70-μm sand, the high content of GT and fine sand might cause DOM retention in the column (Fig. S6). Negatively charged DOM was readily adsorbed on GT during transport, forming a ligand exchange between the carboxyl/hydroxyl functional groups of DOM and the GT surface[17]. The retention of DOM significantly changed the properties of the GT-coated sand. The retention rate was the highest (average: 43.0%) in BC_{DOM} because of its low concentration (Table S6); however, the amount retained in the column was low. The retention rate of WS_{DOM} (average: 36.2%) was higher than that of SM_{DOM} (average: 20.9%) (Table S6). WS_{DOM} was readily deposited in the 2% GT-coated 70-μm sand column (71%) (Fig. S6 and Table S6) because the protein-like substance in DOM promoted the formation of bridged complexes with GT and organic molecules[18], and the small pore structure facilitated this process.

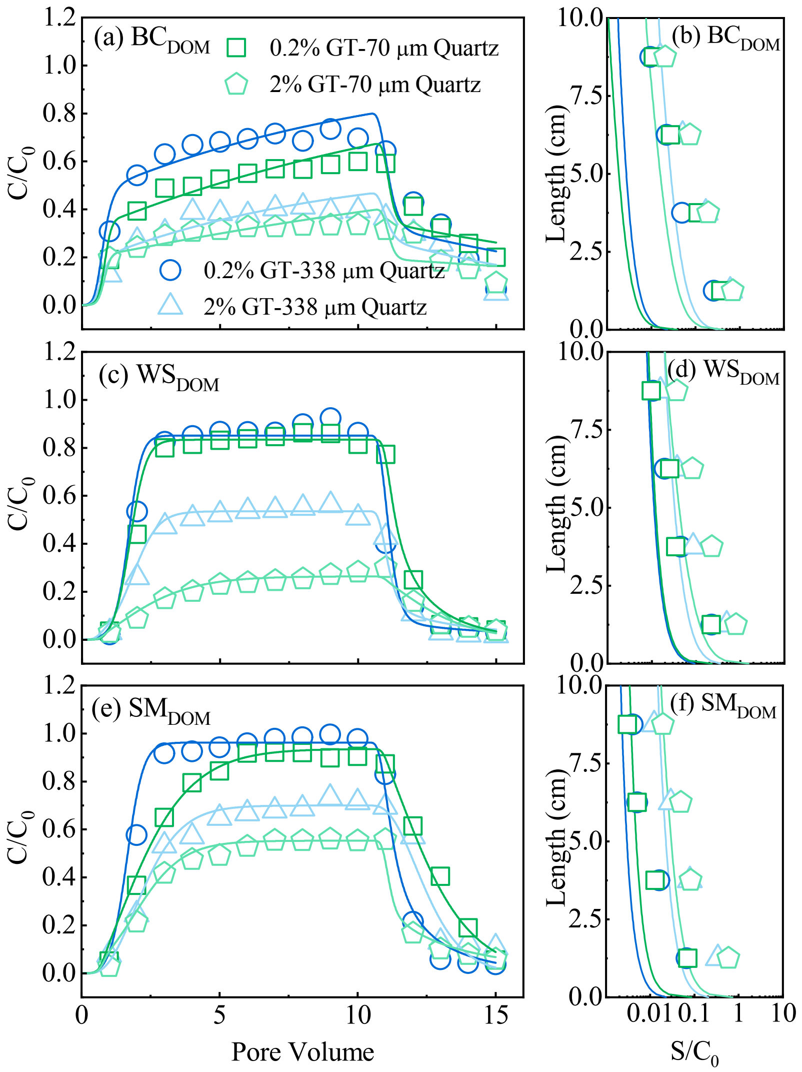

Figure S6. Breakthrough curves (a, c, and e) and RPs (b, d, and f) of BC_{DOM} (a and b), WS_{DOM} (c and d), and SM_{DOM} (e and f) at pH 7.0. The DOM RPs were plotted as the initial DOM concentration normalized (DOM retention in the sands columns S_r divided by initial DOM concentration) as DOM retention per gram of dry sand as a function of distance from the column inlet. Symbols and solid lines show the observed data and simulation fitting, respectively.

S14. Fitted parameters of DOM transport in the GT coated sand columns

Table S6. Fitted parameters of transport of DOM released from BC, WS, and SM in the 0.2% and 2% GT coated 70 μm and 338 μm sand columns at pH 6.0.

DOM	Column	k_{1a} (min^{-1})	k_{1d} (min^{-1})	k_{2a} (min^{-1})	k_{1d}/k_{1a}	R^2	Recovery (%)		
							Effluent	Column	Total
BC_{DOM}	0.2% GT-70 μm Quartz	0.125	0.016	0.005	0.127	0.931	67.52	35.25	102.77
	2% GT-70 μm Quartz	0.143	0.012	0.549	0.082	0.825	40.50	61.98	102.48
	0.2% GT-338 μm Quartz	0.084	0.022	0.027	0.262	0.951	80.22	21.32	101.54
	2% GT-338 μm Quartz	0.129	0.028	0.399	0.217	0.811	45.92	53.40	99.32
WS_{DOM}	0.2% GT-70 μm Quartz	0.537	0.225	0.273	0.418	0.995	83.17	18.30	101.47
	2% GT-70 μm Quartz	0.304	0.186	2.061	0.612	0.954	26.25	70.14	96.39
	0.2% GT-338 μm Quartz	0.603	0.019	0.112	0.032	0.975	81.6	16.79	98.39
	2% GT-338 μm Quartz	0.373	0.045	0.446	0.119	0.990	50.81	39.72	90.53
SM_{DOM}	0.2% GT-70 μm Quartz	0.300	0.200	0.100	0.666	0.994	94.03	7.78	101.81
	2% GT-70 μm Quartz	0.250	0.061	0.900	0.244	0.979	53.32	44.16	97.48
	0.2% GT-338 μm Quartz	0.589	0.206	0.026	0.350	0.994	94.81	6.96	101.77
	2% GT-338 μm Quartz	0.785	0.568	0.251	0.724	0.964	71.74	24.79	96.53

S15. Fitted parameters of nanoplastics co-transport with DOM in the GT coated sand columns

Table S7. Fitted parameters of NPs co-transport with different concentrations DOM released from BC, WS, and SM in the 0.2% and 2% GT coated 70 μm and 338 μm sand columns at pH 6.0.

NPs	Column	DOM	k_{1a} (min^{-1})	k_{1d} (min^{-1})	k_{2a} (min^{-1})	k_{1d}/k_{1a}	R^2	Recovery (%)
50NPs	2% GT-70 μm Quartz	quarter BC_{DOM}	-	-	-	-	-	0.0 ± 0.0
		quarter WS_{DOM}	0.67 ± 0.24	0.308 ± 0.308	1.957 ± 1.956	0.342 ± 0.340	0.823 ± 0.118	8.3 ± 0.1
		quarter SM_{DOM}	-	-	-	-	-	4.0 ± 0.2
	2% GT-338 μm Quartz	quarter BC_{DOM}	0.25 ± 0.04	0.001 ± 0.001	0.025 ± 0.023	0.005±0.004	0.989±0.008	71.5 ± 2.7
		quarter WS_{DOM}	0.18 ± 0.01	0.004 ± 0.002	0.086 ± 0.006	0.023 ± 0.011	0.982 ± 0.01	74.6 ± 0.5
		quarter SM_{DOM}	0.24 ± 0.01	0.002 ± 0.001	0.169 ± 0.018	0.009 ± 0.004	0.983 ± 0.008	54.5 ± 1.3
400NPs	0.2% GT-70 μm Quartz	quarter BC_{DOM}	-	-	-	-	-	0.0 ± 0.0
		quarter WS_{DOM}	-	-	-	-	-	0.8 ± 0.1
		quarter SM_{DOM}	-	-	-	-	-	4.0 ± 0.4
	0.2% GT-338 μm Quartz	quarter BC_{DOM}	0.35 ± 0.02	0.002 ±0.001	1.228 ±0.120	0.005 ±0.004	0.930 ±0.016	10.3 ±0.5
		quarter WS_{DOM}	0.29 ± 0.02	0.007 ± 0.003	1.198 ± 0.065	0.024 ±0.001	0.946 ± 0.001	14.0 ± 0.9
		quarter SM_{DOM}	0.56 ± 0.03	0.000 ± 0.000	0.934 ± 0.143	0.000 ± 0.000	0.892 ± 0.026	14.6 ± 2.4
50NPs	2% GT-70 μm Quartz	half BC_{DOM}	1.04 ± 0.72	0.409 ± 0.174	2.900 ± 0.034	0.532 ±0.200	0.932 ±0.002	15.6 ± 0.1
		half WS_{DOM}	1.41 ± 1.00	0.980 ± 0.980	1.542 ± 1.539	0.406 ± 0.406	0.858 ± 0.074	12.5 ± 1.4
		half SM_{DOM}	-	-	-	-	-	3.4 ± 2.4

	2% GT-338 μm Quartz	half BC_{DOM}	0.37 ± 0.05	0.001 ± 0.000	0.073 ± 0.035	0.002 ± 0.000	0.991 ± 0.003	58.5 ± 2.6
		half WS_{DOM}	0.22 ± 0.01	0.002 ± 0.000	0.144 ± 0.025	0.011 ± 0.000	0.987 ± 0.002	59.3 ± 0.2
		half SM_{DOM}	0.34 ± 0.00	0.002 ± 0.000	0.264 ± 0.061	0.006 ± 0.000	0.981 ± 0.003	41.4 ± 1.9
400NPs	0.2% GT-70 μm Quartz	half BC_{DOM}	1.57 ± 0.64	0.010 ± 0.005	0.394 ± 0.394	0.006 ± 0.001	0.942 ± 0.020	24.3 ± 1.6
		half WS_{DOM}	0.45 ± 0.01	0.009 ± 0.009	2.733 ± 0.346	0.019 ± 0.019	0.907 ± 0.017	7.5 ± 1.2
		half SM_{DOM}	0.481 ± 0.027	0.027 ± 0.004	0.277 ± 0.001	0.057 ± 0.012	0.982 ± 0.002	72.5 ± 1.3
	0.2% GT-338 μm Quartz	half BC_{DOM}	0.32 ± 0.03	0.001 ± 0.000	0.000 ± 0.000	0.003 ± 0.000	0.974 ± 0.001	58.2 ± 3.9
		half WS_{DOM}	0.66 ± 0.13	0.004 ± 0.001	0.001 ± 0.001	0.007 ± 0.003	0.949 ± 0.002	31.9 ± 6.5
		half SM_{DOM}	0.48 ± 0.04	0.014 ± 0.007	0.021 ± 0.004	0.029 ± 0.013	0.980 ± 0.004	83.0 ± 0.2
50NPs	2% GT-70 μm Quartz	pristine BC_{DOM}	0.480 ± 0.150	0.178 ± 0.143	2.910 ± 0.807	0.308 ± 0.203	0.682 ± 0.125	12.3 ± 2.2
		pristine WS_{DOM}	0.208 ± 0.085	0.158 ± 0.056	2.655 ± 0.005	0.780 ± 0.049	0.955 ± 0.006	18.5 ± 0.1
		pristine SM_{DOM}	-	-	-	-	-	2.3 ± 0.1
	2% GT-338 μm Quartz	pristine BC_{DOM}	0.517 ± 0.050	0.000 ± 0.000	0.264 ± 0.019	0.000 ± 0.000	0.924 ± 0.023	47.0 ± 0.4
		pristine WS_{DOM}	0.249 ± 0.001	0.000 ± 0.000	0.372 ± 0.008	0.000 ± 0.000	0.928 ± 0.003	46.7 ± 1.3
		pristine SM_{DOM}	0.369 ± 0.001	0.006 ± 0.001	0.315 ± 0.184	0.015 ± 0.001	0.955 ± 0.015	38.4 ± 3.0
400NPs	0.2% GT-70 μm Quartz	pristine BC_{DOM}	1.362 ± 0.072	0.064 ± 0.020	0.186 ± 0.028	0.048 ± 0.017	0.969 ± 0.007	71.7 ± 3.9
		pristine WS_{DOM}	2.543 ± 0.178	0.010 ± 0.001	0.386 ± 0.037	0.004 ± 0.001	0.901 ± 0.019	29.0 ± 1.4
		pristine SM_{DOM}	0.764 ± 0.059	0.052 ± 0.020	0.262 ± 0.004	0.070 ± 0.032	0.983 ± 0.002	76.7 ± 1.6
	0.2% GT-338 μm Quartz	pristine BC_{DOM}	0.363 ± 0.012	0.024 ± 0.004	0.000 ± 0.000	0.065 ± 0.009	0.965 ± 0.009	84.8 ± 0.2
		pristine WS_{DOM}	0.331 ± 0.007	0.016 ±0 .003	0.002 ± 0.002	0.049 ± 0.008	0.976 ± 0.003	78.8 ± 0.1
		pristine SM_{DOM}	0.802 ± 0.327	0.430 ±0.420	0.043 ± 0.019	0.388 ± 0.365	0.981 ± 0.009	88.2 ± 0.0

S16. Stability of DOM and its influence on the stability of nanoplastics

Settling experiments of individual NPs and pristine DOM and NPs-DOM (with different DOM concentrations) at pH 6.0 were studied. In settling experiments, The influent concentrations of the 50NPs and 400MPs were maintained at 100 mg L^{-1}. Each suspension was immediately transferred into cuvettes for the measurement of absorbance at 300 nm over a period of 24 h. All settling experiments were conducted in duplicate. The settling curves were then plotted, whereby the ordinate was the ratio of the absorbance values at a given point in time (A) to the initial absorbance (A_0) and the abscissa was time.

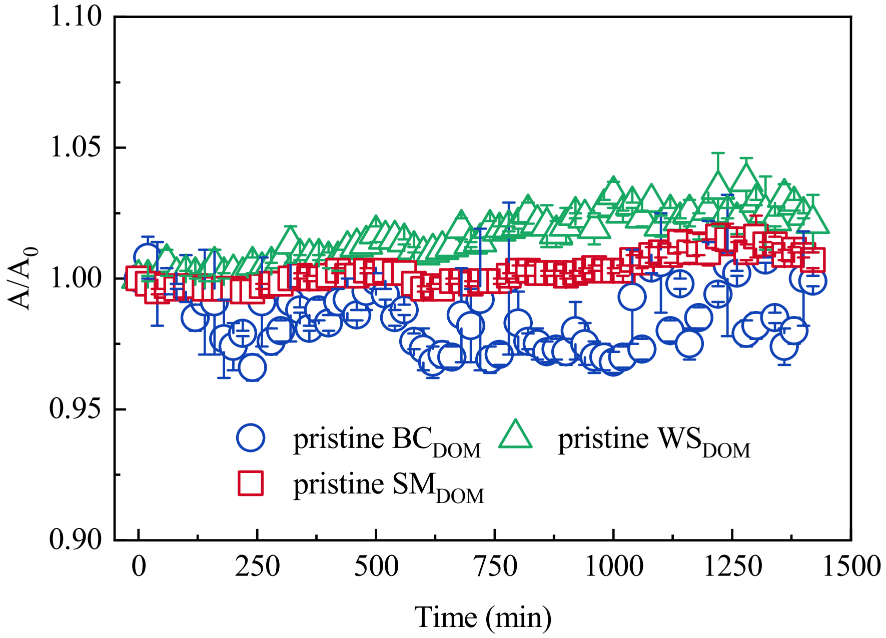

Figure S7. The settling curve of different DOM.

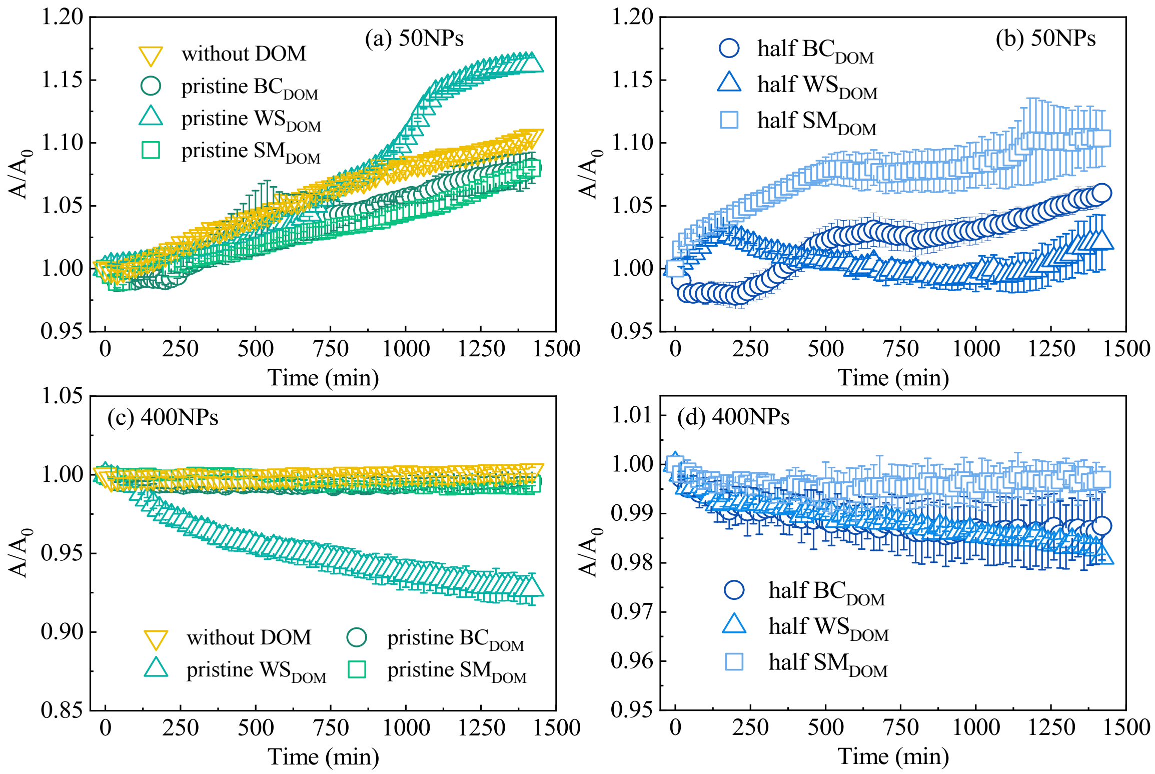

Figure S8. The settling curve of NPs with and without different DOM.

S17. DLVO interaction energy between nanoplastics and GT-coated sand before and after co-transport with DOM

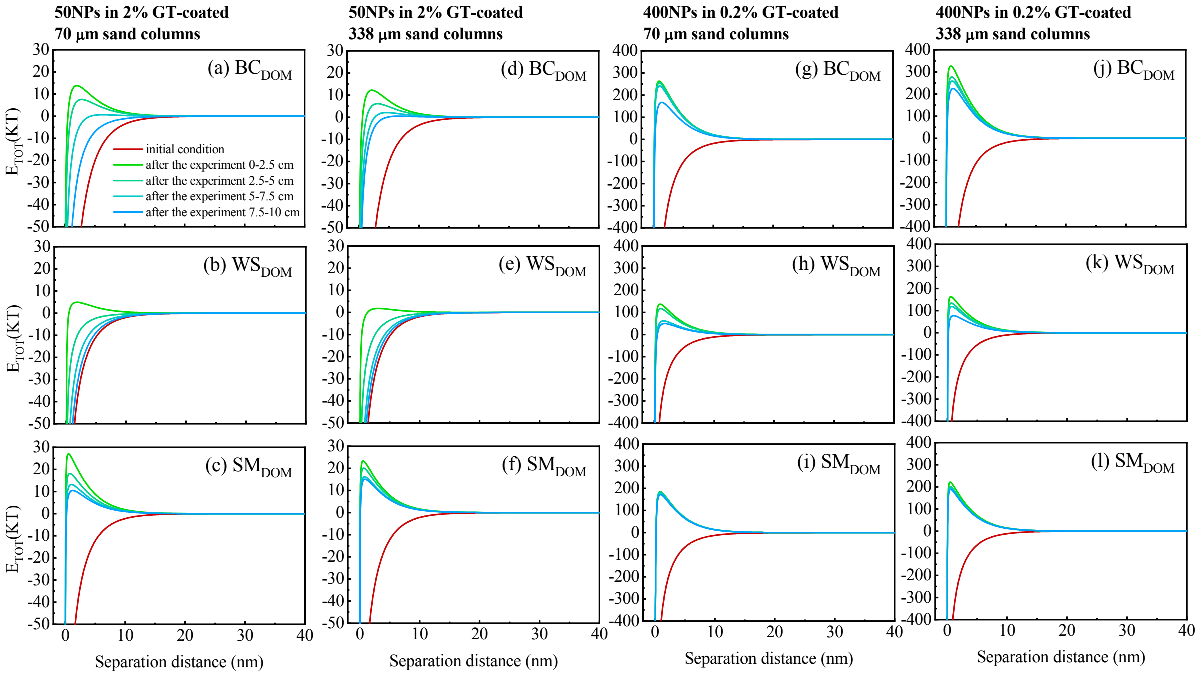

Figure S9. DLVO interaction energy between NPs and sand before (red lines) and after (others) co-transport experiments of NPs and pristine DOM. The E_{TOT} after co-transport experiment were calculated based on four segmented columns (0-2.5, 2.5-5, 5-7.5, and 7.5-10 cm).

S18. Deposition of nanoplastics

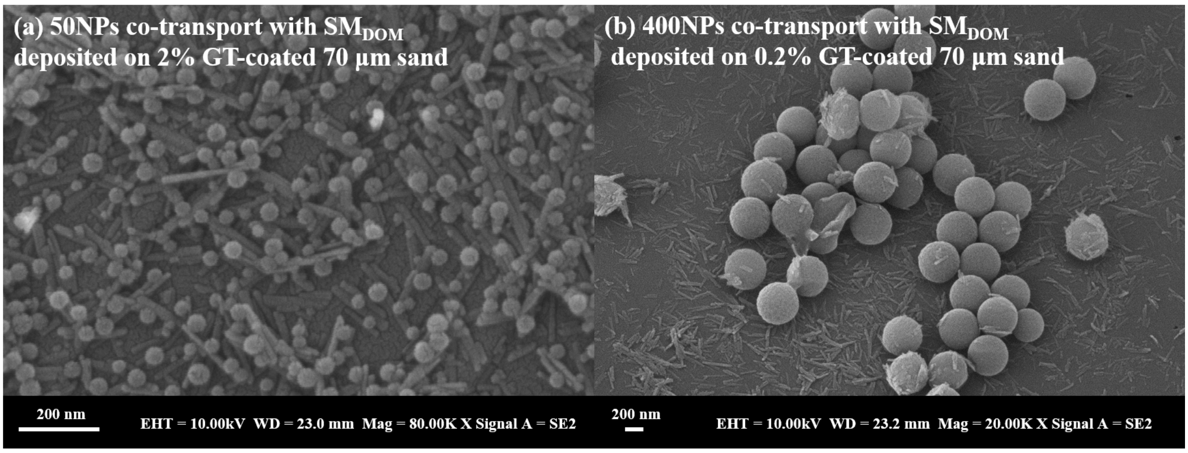

Figure S10. SEM images of NPs deposited on GT coated sand.

S19. XPS results of nanoplastics and different DOM co-deposited on GT coated sand

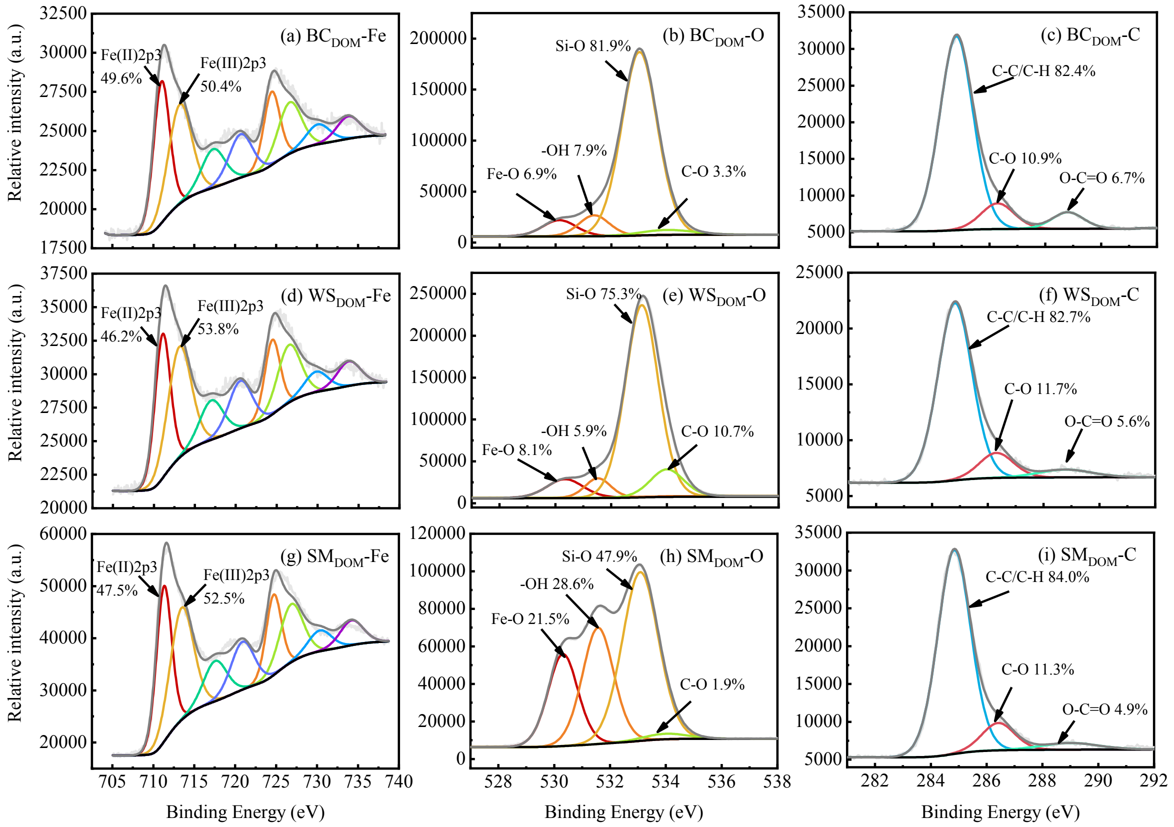

Figure S11. X-ray photoelectron spectroscopy of 50NPs co-transport with different DOM deposited on GT-coated sand. Data was identified by X-ray photoelectron spectroscopy with an Al Kα X-ray source (1486.6 eV). Survey spectra were recorded from 1200 ~ 0 eV for each sample in a vacuum of 8 $\times 10^{-10}$ Pa. All peaks were calibrated using the C1s peak at 284.8 eV. The data was processed using the XPSPEAK 4.1.

S20. Result parameters of quantum chemical computation

Table S8 Area vertex coordinates and mutual penetration distance.

System	Position	Vertex Coordinate 1			Vertex Coordinate 2			Mutual penetration distance (Å)
		x	y	z	x	y	z	
NPs-CL	A	5.72	-0.42	-3.51	6.18	-1.18	-2.94	1.050
	B	-3.69	-0.13	-0.78	-3.41	-1.06	-0.03	1.226
	C	-5.17	-1.35	-2.23	-4.92	-2.27	-1.64	1.128
NPs-AM	A	5.40	-2.81	2.38	5.40	-2.18	2.36	0.635
	B	1.68	-0.75	3.98	1.72	0.06	4.17	0.831
	C	-4.34	-4.01	3.75	-3.31	-3.62	4.34	1.249
NPs-OA	A	3.13	0.99	-3.50	2.92	0.79	-3.70	0.359
	B	1.94	1.26	-1.58	1.83	1.23	-1.74	0.201
	C	0.92	1.83	-2.93	0.94	1.68	-3.01	0.168
NPs-TP	A	2.67	1.10	-3.29	1.75	1.43	-2.76	1.119
	B	0.15	-2.63	-4.23	0.07	-2.39	-4.23	0.252
	C	-3.92	-2.25	-3.92	-3.49	-2.45	-3.02	1.010
NPs-HA	A	6.026	3.116	0.908	5.947	3.355	-0.123	1.061
	B	4.069	3.773	3.078	4.61	2.943	3.586	1.113
	C	1.397	3.62	3.329	1.109	2.664	3.119	1.020
NPs-FA	A	-4.418	3.576	-2.299	-5.249	3.718	-2.993	1.092
	B	-1.521	-2.085	-3.685	-2.034	-3.068	-4.184	1.216
	C	-0.843	-3.903	-3.590	-1.356	-4.375	-3.983	0.800

Table S9 Binding energy between representative DOM and NPs.

System	Binding energy ($kJ\ mol^{-1}$)	Binding energy (Hartree)	$E_{complex}$ (Hartree)	$E_{fragment1}$ (Hartree)	$E_{fragment2}$ (Hartree)
NPs-CL	-178.29	-0.0679	-3685.317	-1240.301	-2444.948
NPs-AM-	-158.609	-0.0604	-3748.058	-1240.290	-2507.707
NPs-OA	-117.76	-0.0449	-2096.255	-1240.302	-855.908
NPs-TP	-121.81	-0.0464	-2420.979	-1240.299	-1180.634
NPs-HA	-169.92	-0.0647	-7059.133	-1240.305	-5818.764
NPs-FA	-166.08	-0.0633	-3639.782	-1240.302	-2399.417

References

1. Chen, W.; Westerhoff, P.; Leenheer, J. A.; Booksh, K., Fluorescence excitation-emission matrix regional integration to quantify spectra for dissolved organic matter. *Environmental Science & Technology* **2003,** *37*, (24), 5701-10.

2. Zsolnay, A.; Baigar, E.; Jimenez, M.; Steinweg, B.; Saccomandi, F., Differentiating with fluorescence spectroscopy the sources of dissolved organic matter in soils subjected to drying. *Chemosphere* **1999,** *38*, (1), 45-50.

3. Yu, B.; Jia, S. Y.; Liu, Y.; Wu, S. H.; Han, X., Mobilization and re-adsorption of arsenate on ferrihydrite and hematite in the presence of oxalate. *Journal of Hazardous Materials* **2013,** *262*, (22), 701-708.

4. Bradford, S. A.; Jirka, S.; Mehdi, B.; Martinus Th, V. G.; Yates, S. R., Modeling colloid attachment, straining, and exclusion in saturated porous media. *Environmental Science & Technology* **2003,** *37*, (10), 2242-50.

5. Derjaguin, B.; Landau, L., Theory of the stability of strongly charged lyophobic sols and of the adhesion of strongly charged particles in solutions of electrolytes. *Acta Physicochim USSR. Band* **1941,** *S*, 30-59.

6. Verwey, E. J. W.; Overbeek, J. T. G., Theory of the stability of lyophobic colloids. *Elsevier, New York* **1948**, OCLC 2313484.

7. Masliyah, J., Particle deposition and aggregation: measurement, modelling and simulation. *Journal of Colloid and Interface Science* **1998,** *200*, (1), 363.

8. Gregory, J., Approximate expressions for retarded van der waals interaction. *Journal of Colloid & Interface Science* **1981,** *83*, (1), 138-145.

9. Israelachvili, J. N., *Intermolecular and surface forces. Academic Press, Cambridge, London, 3rd Edition*. 2011; Vol. 2, p 59–65.

10. Bergström, L., Hamaker constants of inorganic materials. *Advances in Colloid & Interface Science* **1997,** *70*, (1), 125-169.

11. Hogg, R.; Healy, T. W.; Fuerstenau, D. W., Mutual coagulation of colloidal dispersions. *Transactions of the Faraday Society* **1966,** *62*.

12. Walters, J. K., *Particle deposition and aggregation, measurement, modelling and*

simulation. Butterworth-Heinemann, University College, London , 1st Edition. 1996; Vol. 64, p 363.

13. Frisch, M. J.; Trucks, G. W.; Schlegel, H. B.; Scuseria, G. E.; Robb, M. A.; Cheeseman, J. R.; Scalmani, G.; Barone, V.; Petersson, G. A.; Nakatsuji, H.; Li, X.; Caricato, M.; Marenich, A. V.; Bloino, J.; Janesko, B. G.; Gomperts, R.; Mennucci, B.; Hratchian, H. P.; Ortiz, J. V.; Izmaylov, A. F.; Sonnenberg, J. L.; Williams-Young, D.; Ding, F.; Lipparini, F.; Egidi, F.; Goings, J.; Peng, B.; Petrone, A.; Henderson, T.; Ranasinghe, D.; Zakrzewski, V. G.; Gao, J.; Rega, N.; Zheng, G.; Liang, W.; Hada, M.; Ehara, M.; Toyota, K.; Fukuda, R.; Hasegawa, J.; Ishida, M.; Nakajima, T.; Honda, Y.; Kitao, O.; Nakai, H.; Vreven, T.; Throssell, K.; Jr. Montgomery, J. A.; Peralta, J. E.; Ogliaro, F.; Bearpark, M. J.; Heyd, J. J.; Brothers, E. N.; Kudin, K. N.; Staroverov, V. N.; A., K. T.; Kobayashi, R.; Normand, J.; Raghavachari, K.; Rendell, A. P.; Burant, J. C.; Iyengar, S. S.; Tomasi, J.; Cossi, M.; Millam, J. M.; Klene, M.; Adamo, C.; Cammi, R.; Ochterski, J. W.; Martin, R. L.; Morokuma, K.; Farkas, O.; Foresman, J. B.; Fox, D. J. *Gaussian 16, Revision B.01*, Gaussian, Inc., Wallingford CT, 2016.

14. Ouni, Y.; Ghnaya, T.; Montemurro, F.; Abdelly, C.; Lakhdar, A., The role of humic substances in mitigating the harmful effects of soil salinity and improve plant productivity. *International Journal of Plant Production* **2014,** *8(3)*, 353-374.

15. Brunt, K.; Sanders, P.; Rozema, T., The Enzymatic Determination of Starch in Food, Feed and Raw Materials of the Starch Industry. *Starch - Stärke* **1998,** *50*, (10), 413-419.

16. Mongeau, R.; Brassard, R., Determination of Neutral Detergent Fiber in Breakfast Cereals: Pentose, Hemicellulose, Cellulose and Lignin Content. *Journal of Food Science* **1982,** *47*, (2), 550-555.

17. Safiur Rahman, M.; Whalen, M.; Gagnon, G. A., Adsorption of dissolved organic matter (DOM) onto the synthetic iron pipe corrosion scales (goethite and magnetite): Effect of pH. *Chemical Engineering Journal* **2013,** *234*, 149-157.

18. Bai, L.; Cao, C.; Wang, C.; Wang, C.; Zhang, H.; Jiang, H., Roles of phytoplankton- and macrophyte-derived dissolved organic matter in sulfamethazine adsorption on goethite. *Environmental pollution* **2017,** *230*, 87-95.
